# Genomic sequence analyses of classical and non-classical lamprey progesterone receptor genes and the inference of homologous gene evolution in metazoans

**DOI:** 10.1186/s12862-019-1463-7

**Published:** 2019-07-01

**Authors:** Jianfeng Ren, Yu-Wen Chung-Davidson, Liang Jia, Weiming Li

**Affiliations:** 1 International Research Center for Marine Biosciences, Ministry of Science and Technology, Shanghai Ocean University, Shanghai, 201306 China; 2 Department of Fisheries and Wildlife, Michigan State University, East Lansing, MI 48824 USA; 3 Key Laboratory of Exploration and Utilization of Aquatic Genetic Resources, Ministry of Education, Shanghai Ocean University, Shanghai, 201306 China; 4 Key Laboratory of Freshwater Aquatic Genetic Resources, Ministry of Agriculture, Shanghai Ocean University, Shanghai, 201306 China

**Keywords:** Nuclear progesterone receptor, Membrane progesterone receptor, Membrane associated progesterone receptor, Lamprey, Metazoan, Evolution

## Abstract

**Background:**

Nuclear progesterone receptor (nPR) is an evolutionary innovation in vertebrates that mediates genomic responses to progesterone. Vertebrates also respond to progesterone via membrane progesterone receptors (mPRs) or membrane associated progesterone receptors (MAPRs) through rapid nongenomic mechanisms. Lampreys are extant agnathan vertebrates, residing at the evolutionary juncture where vertebrates diverged from invertebrates. A survey of the progesterone receptor (PR) gene sequences in lamprey genomes would inform PR gene evolutionary events during the transition from invertebrates to vertebrates.

**Results:**

In this study, we annotated sequences of one nPR, four mPR (β, γ, δ and ε) and four MAPR genes from genomes of two lamprey species (*Petromyzon marinus* and *Lethenteron japonicum*). To infer the origin and evolutionary history of PR genes, we constructed phylogenetic trees of PR homologous sequences across representative species of metazoans. Phylogenetic analyses revealed that the mPRγ gene first appeared in non-bilaterians, and the mPRβ gene likely arose from a duplication of mPRγ. On the other hand, the mPRγ gene gave rise to the mPRδ and ε genes much later in the vertebrate lineage. In addition, the mPRα gene first appeared in cartilaginous fishes, likely derived from duplication of mPRβ after the agnathan-gnathostome divergence. All known MAPR genes were present in the lamprey genomes. Progesterone receptor membrane component 1 (PGRMC1), neudesin and neuferricin genes probably evolved in parallel in non-bilaterians, whereas two copies of PGRMC genes probably derived from duplication of ancestral PGRMC1 sequence and appeared before the speciation of lampreys.

**Conclusions:**

Non-classical mPR and MAPR genes first evolved in non-bilaterians and classical nPR genes evolved later in basal vertebrates. Sequence repertoires for membrane progesterone receptor genes in vertebrates likely originated from an ancestral metazoan sequence and expanded via several duplication events.

**Electronic supplementary material:**

The online version of this article (10.1186/s12862-019-1463-7) contains supplementary material, which is available to authorized users.

## Background

Progesterone (P_4_) is one of the first discovered steroid hormones with known functions and subsequently pursued as a drug target [1]. It was initially discovered as a sex hormone, and later found to have broader effects, ranging from inhibition of apoptosis to regulation of cholesterol biosynthesis and axon guidance [2, 3]. Classical studies of P_4_ signaling have been focused on nPR, a P_4_-activated transcription factor that directly regulates gene expression. More recently, P_4_ signaling has been noted for its role in modulating diverse neural processes such as cognitive functions and emotions [4, 5], neurogenesis [6, 7], neuroinflammation [8], neuroprotection and neuroplasticity [9–11]. It is now recognized that P_4_ acts through both classical nPR that elicits slower genomic responses, and the non-classical membrane receptors that exert fast-acting non-genomic responses [12].

Non-classical PRs consist of mPRs [13] and MAPRs [14]. The mPRs are G-protein coupled receptors (GPCRs) that belong to Class II progestin and adipoQ receptor (PAQR) family, including mPRα (PAQR7), mPRβ (PAQR8), mPRγ (PAQR5), mPRδ (PAQR6), and mPRε (PAQR9) [13]. mPRα was first characterized and isolated from the membrane fractions of seatrout ovary [15]. Subsequently, mPRα, β and γ were isolated from human, mouse, pig and other vertebrates [16, 17], with highly specific P_4_ binding abilities [13, 16]. The proteins encoded by mPRα, β and γ genes are typically coupled to a pertussis-sensitive inhibitory G protein (G_i_) that inhibits adenylyl cyclase activity, resulting in a decrease in secondary messenger cAMP levels [18]. On the contrary, mPRδ and ε are coupled to a stimulatory G protein (G_s_) that activates adenylyl cyclase and increases cAMP levels [19]. It is now widely accepted that mPRs have specific and saturable high-affinity binding sites for P_4_ [19].

The MAPRs include PGRMC1 [2, 3], PGRMC2 [20], neudesin [21], and neuferricin [22]. PGRMC1 was first isolated from porcine liver cell membranes with high P_4_-binding affinity [23]. Recent studies showed that P_4_ binds to human PGRMC1 [24], which acts as an adaptor protein of mPR to form a mPR protein complex [25]. PGRMC2 is structurally similar to PGRMC1 and likely evolved from a common ancestor [3, 26]. Neudesin (neudesin neurotrophic factor, NENF) was first characterized as a neurotrophic factor [21], whereas neuferricin (cytochrome b5 domain containing 2, CYB5D2) is structurally related to neudesin, with similar cytochrome b5 (cyt-b5)-like heme-binding domain [22]. Unlike PGRMC1 and PGRMC2, neudesin and neuferricin appear to be secretory proteins [22], with no P_4_-binding ability [27, 28]. It remains unclear whether the other MAPR members are involved in formation of the mPR protein complex, or exhibit P_4_ binding ability as PGRMC1.

So far, nPR genes have only been found in vertebrates [29]. By contrast, an ortholog of PR exists in invertebrate cephalochordates (also known as lancelets or amphioxi) [30, 31], and DNA sequences homologous to mPR or MAPR genes are more widespread in invertebrates, bacteria and fungi [32–35]. The endogenous hormonal function of P_4_ via nPR in vertebrates likely evolved in parallel with the neuroendocrine hypothalamic-pituitary-gonadal axis [36]. On the other hand, the sporadic occurrence of progestins, their biosynthetic enzymes and functional receptors in invertebrates [37, 38], suggests that these mPRs or MAPRs were not yet co-opted to bind endogenous progestins, or they likely responded to exogenous progestins produced by other organisms equipped with a full suite of biosynthetic enzymes [39]. Regardless of their actual ligands, these homologous sequences provided the foundation for the evolution of vertebrate mPR or MAPR genes.

Lampreys represent the basal jawless vertebrate lineage that diverged from jawed vertebrates around 560 million years ago [40, 41]. Sea lamprey possess a nPR gene [29]. There is evidence of membrane progesterone-binding activity but no specific receptor proteins or genes have been identified in sea lamprey [42]. The release of the draft genomes of two lamprey species, sea lamprey *Petromyzon marinus* and Japanese lamprey *Lethenteron japonicum* [43, 44], makes viable a genomic survey for different PR genes and the results may shed light on PR gene evolution from invertebrates to vertebrates. In this study, we sought to identify PR genes in lamprey genomes and to investigate the origin and evolutionary history of homologous gene sequences in metazoans through phylogenetic analyses.

## Results

### Characteristics of lamprey nPR gene sequence

One nPR gene was identified in the sea lamprey and Japanese lamprey genomes. The transcript, coding DNA sequence (CDS), and predicted amino acid sequence are listed in Table 1. The CDS region of sea lamprey nPR is 12 bp longer than that of Japanese lamprey. The nucleotide and amino acid residue sequences are 96.7 and 98.0% identical between the sea lamprey and Japanese lamprey.

**Table 1:** Detailed information for nPR, mPR and MAPR gene families identified in lampreys

Gene/transcript name	Sea lamprey					Japanese lamprey			Domains	Reference^b^ (aa)
	GenBank accession No.	Transcript (bp)	CDS (bp)	Protein (aa)	CDS status^a^	CDS (bp)	Protein (aa)	CDS status		
nPR	KT970662	1967	1341	446	C	1329	442	C	DBD/LBD	933
mPRβ isoform1	KT970648	2016	1077	358	C	1077	358	C	TM/HlyIII	354
mPRβ isoform2	KT970649	2038	1077	358	C	–	–	–	TM/HlyIII	354
mPRβ isoform3	KT970650	2324	1077	358	C	–	–	–	TM/HlyIII	354
mPRβ isoform4	KT970651	2593	1077	358	C	–	–	–	TM/HlyIII	354
mPRγ	KT970652	1621	1089	362	C	1080	359	C	TM/HlyIII	330
mPRδ isoform1	KT970653	1984	1029	342	C	1029	342	C	TM/HlyIII	341
mPRδ isoform2	KT970654	1273	873	290	C	–	–	–	TM/HlyIII	341
mPRδ isoform3	KT970655	1786	873	290	C	–	–	–	TM/HlyIII	341
mPRδ isoform4	KT970656	2236	873	290	C	–	–	–	TM/HlyIII	341
mPRε	KT970657	882	822	273	P	1023	340	C	TM/HlyIII	377
PGRMC1	KT970658	1485	552	183	C	549	182	C	TM/Cyt-b5	195
PGRMC2	KT970659	689	417	138	P	396	131	P	TM/Cyt-b5	247
neudesin	KT970660	609	525	174	C	525	174	C	SP/Cytb-b5	172
neuferricin	KT970661	1592	840	279	C	849	282	C	SP/Cytb-b5	264

^a^P indicates partial sequence, C indicates complete sequence. ^b^Reference indicates the length of the proteins in human

**Fig. 1:**
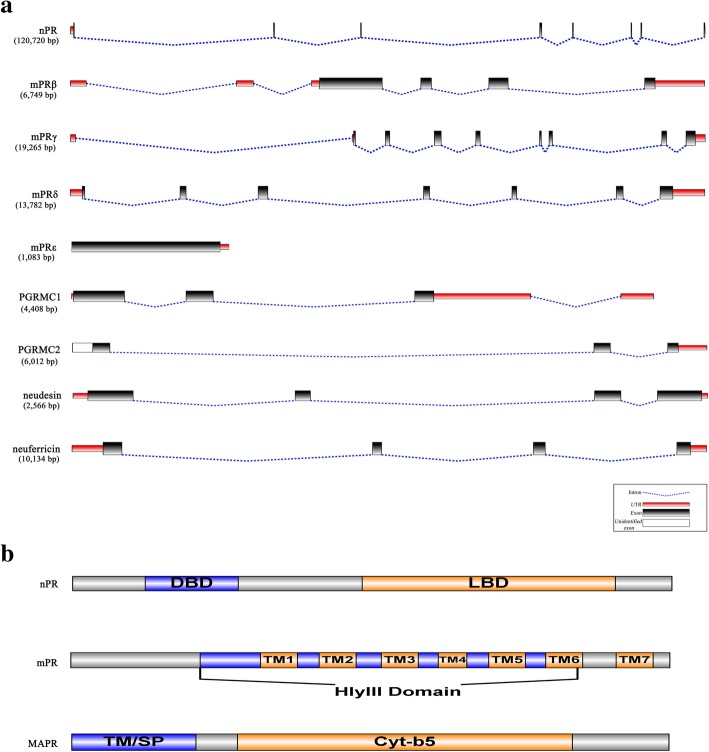
Schematic diagrams of the gene structure (**a**) and protein domains (**b**) for nPR, mPR and MAPR gene families. The number in the parenthesis indicates the length of genes spanned on the chromosome. TM/SP represents either the transmembrane domain present in PGRMC1 and PGRMC2 or the signal peptide present in neudesin and neuferricin. DBD: DNA-binding domain, LBD: ligand-binding domain, TM: transmembrane, HlyIII: haemolysin-III, SP: signal peptide, and Cyt-b5: cytochrome b5-like heme/steroid binding domain

The lamprey nPR gene consisted of 8 exons and spanned 120,720 bp on the chromosome (Fig. 1a), consistent with those in human, mouse and zebrafish (Additional file 3). The 12 additional bp of the sea lamprey nPR gene were located in the fourth exon of the CDS region. Protein domain structure analysis showed that the predicted nPR protein contained two highly-conserved domains, the DNA-binding domain (DBD) and the ligand-binding domain (LBD) (Fig. 1b). The DBD consisted of approximately 70 amino acids (55–124) with two C4-type zinc fingers, responsible for the interaction between the hormone-receptor complex and the hormone response element, located within the promoter region of the target genes [45]. The LBD was located at the carboxyl terminus (216–404), containing sub-regions for ligand binding, dimerization and transcriptional regulation [46].

Protein motif analyses revealed that the lamprey nPR protein contained Erk1 kinase and Itk Src homolog 2 (SH2) domains that were not found in lamprey mPR or MAPR sequences (Table 2, Additional file 4). Nonetheless, similar domains for SH2 or SH3 interactions, kinase or kinase-binding were present in mPR or MAPR sequences. These findings indicate that mPRs, MAPRs and nPR may potentially recognize and interact with similar proteins in lampreys.

**Table 2:** Motif frequency from lamprey progesterone receptor protein sequences

Motif group	Motif name	mPRβ	mPRγ	mPRδ	mPRε	PGRMC1	PGRMC2	neudesin	neuferricin	nPR
Acidophilic Ser/Thr kinase	Casein kinase 1	0	0	0	0	1^a^	0	0	0	0
	Casein kinase 2	0	0	0	0	2	1^a^	0	0	0
	GSK3	2	0	0	0	0	0	0	0	0
Basophilic Ser/Thr kinase	Akt kinase	0	0	0	0	0	1	0	1	1
	AMP kinase	1^a^	0	2	0	0	0	0	0	1
	Calmodulin dependent kinase 2	0	0	0	0	0	0	0	1	0
	Clk2 kinase	1	0	0	1	0	0	0	0	0
	PKC_δ_	1	1	1	0	0	0	0	0	0
	PKC_ε_	0	0	0	0	0	0	1^a^	0	1
	PKC_ζ_	2	0	0	1	0	0	0	0	0
	PKC_μ_	0	0	1	0	0	0	0	0	1
DNA damage kinase	DNA PK	0	0	0	0	0	2	1^a^	0	2
	ATM kinase	0	0	1	0	0	0	1^a^	0	1^a^
Kinase binding site	Erk1 binding	1^a^	2 (1^a^)	2	0	0	0	0	0	0
	Erk D-domain	2	0	0	1	0	1	1	2	0
	PDK1 binding	1	1	0	2 (1^a^)	0	0	0	1	2
Lipid binding	PIP_3_ binding PH	0	2	0	0	0	0	0	0	0
Phospho-Ser/Thr binding	14-3-3 mode 1	0	1	0	0	0	0	0	0	1
Proline-dependent Ser/Thr kinase	Erk1 kinase	0	0	0	0	0	0	0	0	1^a^
Src homology 2 (SH2)	Abl SH2	0	1	0	0	0	0	0	0	0
	Grb2 SH2	0	0	0	1	0	0	0	0	0
	Itk SH2	0	0	0	0	0	0	0	0	1
	PLCγ C-terminal SH2	0	1	0	0	0	0	0	0	0
	Shc SH2	0	0	0	0	0	1	0	0	1
	SHIP SH2	0	0	0	0	0	1	0	0	0
Src homology 3 (SH3)	Cbl-associated protein C-SH3	1^a^	0	0	0	0	0	0	0	0
	Grb2 SH3	0	0	0	0	0	0	0	1	0
	Itk SH3	1	0	0	0	0	0	0	0	0
	p85 SH3 mode 2	1	0	0	0	0	0	0	0	0
Tyrosine kinase	Abl kinase	0	0	0	0	1	0	0	0	0
	Insulin receptor kinase	1	0	0	0	0	0	0	0	0
	Lck kinase	0	0	0	0	1	1	0	0	1
	Src kinase	0	0	0	0	0	1	0	0	1

Protein motifs were predicted using the Scansite cell signaling interactions prediction “MotifScan” module (https://scansite4.mit.edu/4.0/#scanProtein) under medium stringency setting. ^a^Motifs that were also predicted under high stringency setting. Motif sequences are listed in Additional file 4

### Phylogenetic analyses of nPR genes and metazoan ER/ERR/AncSR genes

**Fig. 2:**
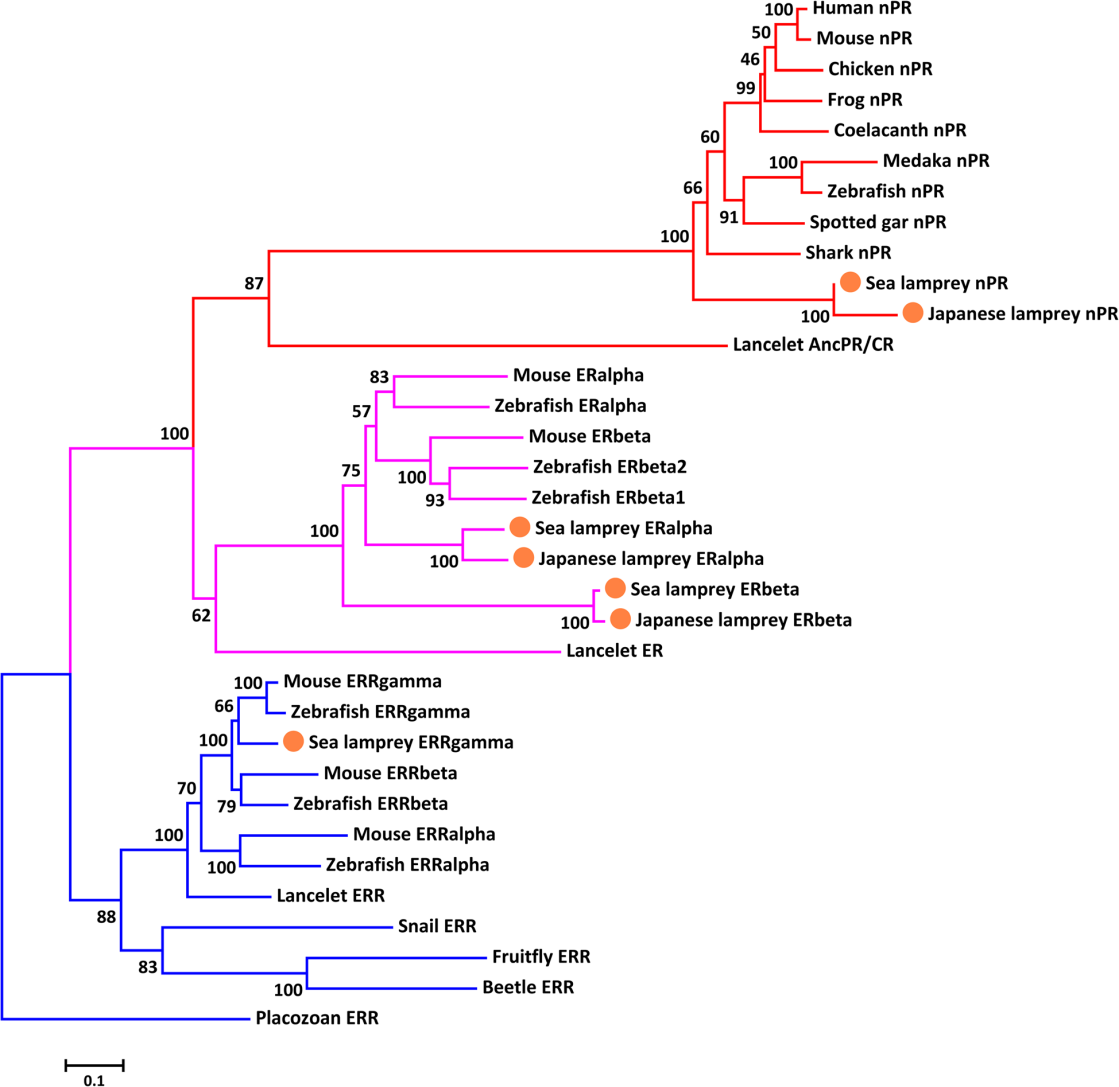
A phylogenetic tree demonstrating the evolutionary relationship between nPR, ER and ERR in metazoans. ERR of placozoan (*Trichoplax adhaerens*) was chosen as the out group. The tree was constructed using the neighbor joining (NJ) method with 100 bootstraps. The blue and pink branches represent the ERR and ER genes appeared in both invertebrates and vertebrates. The red branch represents the nPR genes appeared in the vertebrate lineage and lancelet. The genes identified in lampreys are highlighted with orange dots

Since the nPR gene is thought to have derived from an ancestral ER gene [29, 47], we performed phylogenetic analysis of nPR, ER and ERR genes from representative metazoan groups to infer the evolutionary position of the lamprey nPR gene. ERR shared a common ancestor with ER [47], and was selected as the out group for the phylogenetic analysis. As expected, nPR genes were only found in vertebrates, and each vertebrate species possessed only one copy of the nPR gene. The phylogenetic trees from both NJ and ML methods were clustered into nPR, ER and ERR clades with the placozoan ERR gene at the base (Fig. 2 and Additional file 5). The lancelet ancestral progesterone/corticoid receptor (the same sequence as AncSR2 in [29]) was at the base of the nPR clade [47]. In general, the selected nPR, ER, and ERR genes were clustered in a pattern consistent with the phylogenetic relationship of the species.

### Characteristics of lamprey mPR genes

Four lamprey mPR genes (mPRβ, γ, δ, and ε) were located in different scaffolds (Additional file 6) in both sea lamprey and Japanese lamprey genomes (Table 1). All genes contained full-length CDSs except sea lamprey mPRε (Table 1). No ortholog of the gnathostome mPRα gene was found in either lamprey genome assembly.

Four mPRβ transcript isoforms were identified in the sea lamprey (Additional file 7), with lengths varying from 2016 to 2593 bp due to variations at the 5′ untranslated region (5’UTR). The CDSs were identical (1077 bp) and encoded 358 amino acids. Contrary to the mPRβ CDS transcribed from a single exon in human, mouse and zebrafish (Additional file 3), lamprey mPRβ CDS was transcribed from four exons and the gene spanned 6749 bp on the chromosome (Fig. 1a). However, the predicted lamprey mPRβ protein length was close to that of human, mouse and zebrafish.

The predicted sea lamprey mPRγ protein (362 amino acids) was 3 amino acids longer than that of the Japanese lamprey, and slightly longer than those in human, mouse and zebrafish. Lamprey mPRγ CDS was transcribed from eight exons, similar to those in zebrafish but in contrast to the seven exons of human and mouse mPRγ (Additional file 3). Sea lamprey mPRγ gene was the longest among the mPR genes, spanning 19,265 bp on the chromosome (Fig. 1a).

Four mPRδ transcript isoforms were identified in the sea lamprey, of which isoforms 2, 3, and 4 possessed the same CDS but differed in the untranslated regions (Table 1 and Additional file 8). The gene sequence for isoform 1 spanned 13,782 bp on the chromosome (Fig. 1a). The structure of the CDS and the amino acid length of isoform 1 were similar to the dominant mPRδ isoform in human, mouse and zebrafish (Additional file 3). Two lamprey mPRδ protein isoforms contained 342 and 290 amino acids, with different amino acid residues at the C-terminus.

The Japanese lamprey mPRε sequence contained a full length CDS while the sea lamprey CDS was incomplete, lacking 67 amino acid residues at the N-terminus (Table 1). Both CDSs were transcribed from a single exon gene, similar to the gene of mPRε in human and mouse, but differed from that of zebrafish in which two exons are separated by an 89 bp intron (Additional file 3).

In general, the predicted lamprey mPR proteins contained 340–359 amino acid residues. These receptors all contained seven trans-membrane (7TM) domains and one highly conserved haemolysin-III (Hly III)-related domain (Fig. 1b). Protein motif analyses revealed that lamprey mPR proteins contained various SH2, SH3, kinase or kinase-binding domains (Table 2). For example, mPRβ contained an insulin receptor kinase domain that was not found in other lamprey PRs, and PIP_3_ binding domain was only found in mPRγ (Table 2).

### Phylogenetic analyses of metazoan mPR sequences

**Fig. 3:**
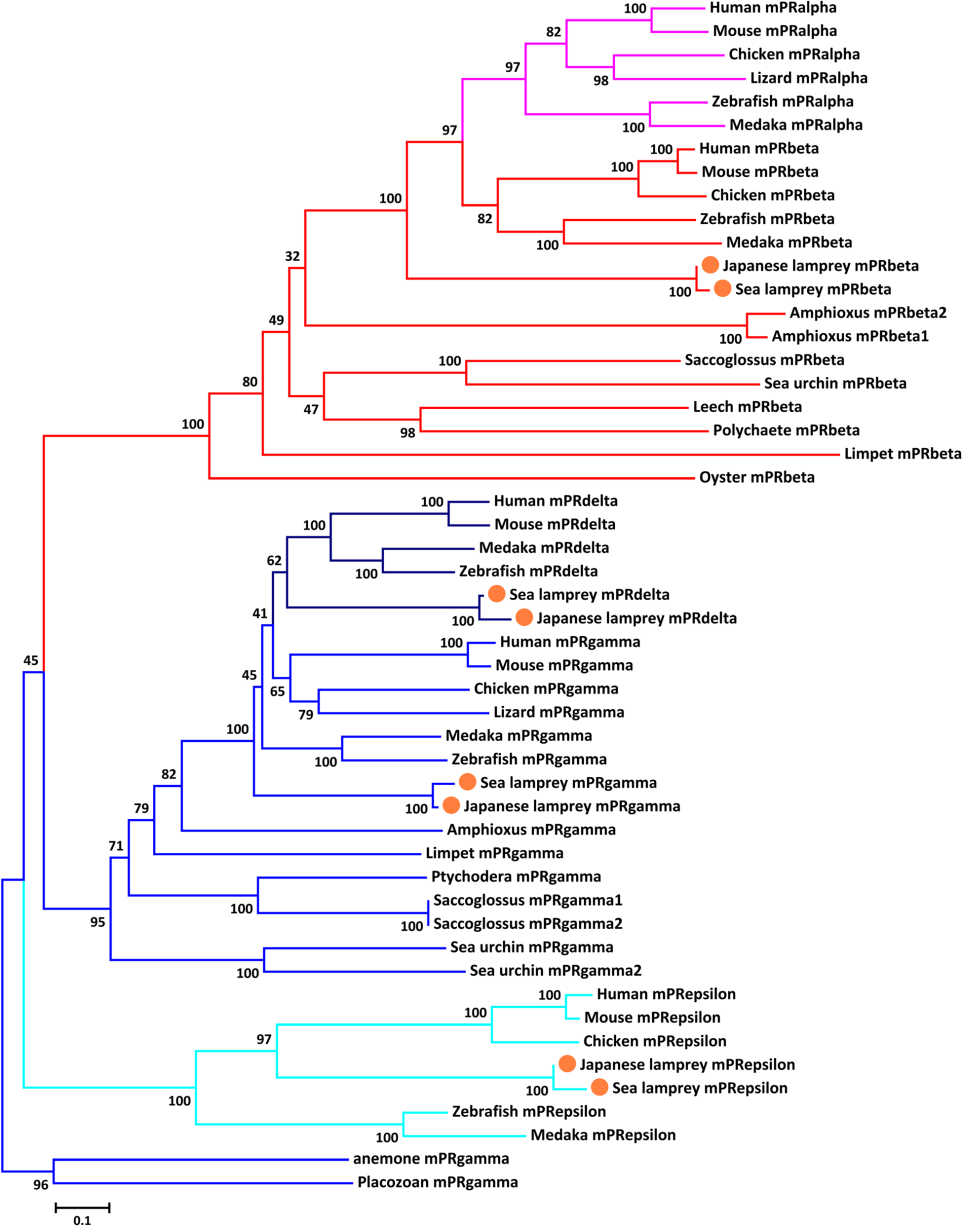
A phylogenetic tree demonstrating the evolutionary relationship among five members of mPR gene families in metazoans. The mPRγ of placozoan and sea anemone were chosen as the out groups. The tree was constructed using the NJ method with 100 bootstraps. Different members of mPR gene family in metazoans are clustered and highlighted with different colors  (pink: mPRα; red: mPRβ; dark blue: mPRδ; blue: mPRγ; light blue: mPRε). The genes identified in lampreys are indicated with orange dots

The mPR gene family expanded in metazoans and increased from one (mPRγ) to five family members (mPRα, β, γ, δ and ε; Table 3). The phylogenetic analyses of mPR genes from representative species using both NJ and ML methods demonstrated the evolutionary relationships among these five genes in metazoans (Fig. 3 and Additional file 9). Placozoan and sea anemone mPRγ genes were positioned at the base of the metazoan lineage. The phylogenetic trees formed three subclades (from bottom to top): vertebrate mPRε, sister groups of bilaterian mPRγ and vertebrate mPRδ, and mPRβ-like genes including mPRα and β (Fig. 3 and Additional file 9). Ancestral mPRγ genes gave rise to mPRβ and mPRγ genes early in bilaterian evolution. On the other hand, the ancestral mPRβ gene further diverged into the “sister” genes mPRα and mPRβ, indicating that mPRα separated from mPRβ gene duplication after the divergence of the agnathans and gnathostomes (Fig. 3 and Additional file 9). In addition, vertebrate mPRδ and mPRγ genes were clustered together in the phylogenetic tree excluding the lamprey mPRγ gene, indicating that the ancestral mPRγ gene divided into mPRγ and δ before the speciation of vertebrates (Fig. 3; also see Table 3). Unfortunately, the origin of the mPRε gene was not properly resolved in the phylogenetic trees. Vertebrate mPRε genes were clustered with mPRγ sequences of early-branching animals, probably resulting from random clustering during phylogenetic construction. Therefore, the mPRε gene likely originated from the mPRγ gene in eumetazoa and was lost in invertebrates, or alternatively arose from gene duplication of mPRγ prior to vertebrate radiation.

**Table 3:** Gene number for nPR, mPR and MAPR gene families identified in representative species

Taxonomy	Common name	Scientific name	mPRα	mPRβ	mPRγ	mPRδ	mPRε	PGRMC1	PGRMC2	neudesin	neuferricin	nPR
Mammalia	Human	*Homo sapiens*	1	1	1	1	1	1	1	1	1	1
Mammalia	Mouse	*Mus musculus*	1	1	1	1	1	1	1	1	1	1
Aves	Chicken	*Gallus gallus*	1	1	1	0	1	1	1	1	1	1
Reptilia	Lizard	*Anolis carolinensis*	1	0	1	0	0	1	1	1	0	1
Amphibia	Frog	*Xenopus tropicalis*	0	1	1	1	1	1	1	1	1	1
Osteichthyes	Coelacanth	*Latimeria chalumnae*	1	1	1	1	1	1	2	1	1	1
Osteichthyes	Takifugu	*Takifugu rubripes*	3	2	1	1	1	1	1	1	1	1
Osteichthyes	Medaka	*Oryzias latipes*	2	1	1	1	1	1	1	1	1	1
Osteichthyes	Zebrafish	*Danio rerio*	2	1	2	1	1	1	1	1	1	1
Osteichthyes	Spotted gar	*Lepisosteus oculatus*	2	1	1	1	1	1	1	1	1	1
Chondrichthyes	Elephant shark	*Callorhinchus milii*	1	0	1	0	0	1	1	1	1	1
Agnatha	Sea lamprey	*Petromyzon marinus*	0	1	1	1	1	1	1	1	1	1
Agnatha	Japanese lamprey	*Lethenteron japonicum*	0	1	1	1	1	1	1	1	1	1
Urochordata	Transparent Sea squirt	*Ciona savignyi*	0	2	2	0	0	0	0	0	1	0
Urochordata	Vase tunicate	*Ciona intestinalis*	0	2	2	0	0	1	0	0	1	0
Cephalochordata	Florida lancelet	*Branchiostoma floridae*	0	8	1	0	0	1	0	1	2	0
Hemichordata	Acorn worm	*Saccoglossus kowalevskii*	0	1	2	0	0	1	0	1	1	0
Echinodermata	Purple sea urchin	*Strongylocentrotus purpuratus*	0	1	3	0	0	1	0	1	2	0
Platyhelminthes	Blood fluke	*Schistosoma mansoni*	0	0	1	0	0	1	0	0	0	0
Annelida	Polychaete worm	*Capitella teleta*	0	2	2	0	0	3	0	1	1	0
Mollusca	Owl limpet	*Lottia gigantea*	0	4	1	0	0	1	0	1	1	0
Mollusca	Pacific oyster	*Crassostrea gigas*	0	3	1	0	0	1	0	0	1	0
Nematoda	Nematode	*Caenorhabditis elegans*	0	1	0	0	0	1	0	0	1	0
Crustacea	Water flea	*Daphnia pulex*	0	0	1	0	0	1	0	0	1	0
Insecta	Fruit fly	*Drosophila melanogaster*	0	0	0	0	0	3	0	0	1	0
Insecta	Silkworm	*Bombyx mori*	0	0	0	0	0	1	0	0	1	0
Cnidaria	Starlet sea anemone	*Nematostella vectensis*	0	0	7	0	0	2	0	1	2	0
Placozoa	Placozoan	*Trichoplax adhaerens*	0	0	3	0	0	1	0	0	1	0
Porifera	Sponge	*Amphimedon queenslandica*	0	0	1	0	0	0	0	0	1	0
Protist	Choanoflagellate	*Monosiga brevicollis*	0	0	0	0	0	1	0	0	1	0

Results of protein motif analyses were consistent with those from phylogenetic analyses (Table 2). Both mPRβ and mPRγ proteins contained basophilic serine/threonine kinase and kinase binding domains. However, mPRβ also acquired SH3 and tyrosine kinase domains, whereas mPRγ acquired PIP_3_-binding and SH2 domains, and mPRδ acquired extra DNA damage kinase domains (Table 2).

### Characteristics of lamprey MAPR genes

Sequences of MAPR gene family (PGRMC1, PGRMC2, neudesin, and neuferricin) were located in different scaffolds (Additional file 6) of sea lamprey and Japanese lamprey genomes. All identified lamprey MAPR genes contained full-length CDSs except PGRMC2. Sea lamprey PGRMC1 gene spanned 4408 bp on the chromosome, and the encoded protein was one amino acid longer than that of Japanese lamprey (Table 1). Lamprey PGRMC1 CDS was transcribed from three exons (Fig. 1a), and the predicted protein length was close to those in human, mouse, and zebrafish (Additional file 3).

Both lamprey PGRMC2 sequences contained incomplete CDSs. Sea lamprey PGRMC2 was longer than that of Japanese lamprey (Table 1). Both predicted proteins were truncated at the N-terminus and were shorter than full-length PGRMC2 proteins in human, mouse and zebrafish. The structures of lamprey PGRMC2 CDSs were similar to those of human, mouse and zebrafish with three exons (Additional file 3), with the missing amino acid sequence encoded in the first exon region (Fig. 1a).

The sea lamprey neudesin gene spanned 2566 bp and contained 5′ and 3′ UTRs (Fig. 1a). The CDS encoded 174 amino acids transcribed from four exons in both lampreys, similar to their orthologs in human, mouse and zebrafish. On the contrary, sea lamprey neuferricin protein was 3 amino acids shorter than that of Japanese lamprey (Table 1). Both lamprey neuferricin CDSs were transcribed from four exons (similar to human, mouse and zebrafish CDSs), but the protein lengths were slightly longer (Additional file 3).

The MAPR proteins identified in lampreys were around 131–282 amino acids long, shorter than lamprey mPR proteins. The MAPR proteins contained either one transmembrane domain (PGRMC1 and PGRMC2) or a signal peptide (neudesin and neuferricin) at the N-terminus, and a heme-binding domain (Cyt-b5) at the C-terminus (Fig. 1b). Protein motif analyses revealed that each MAPR protein contained unique domains, but also retained similar domains that might interact in overlapping signal transduction pathways. For example, neuferricin contained a calmodulin-dependent kinase 2 domain, PGRMC1 possessed an Abl kinase domain, and both PGRMC1 and 2 contained casein kinase and LSK kinase domains (Table 2).

### Phylogenetic analyses of metazoan MAPR sequences

**Fig. 4:**
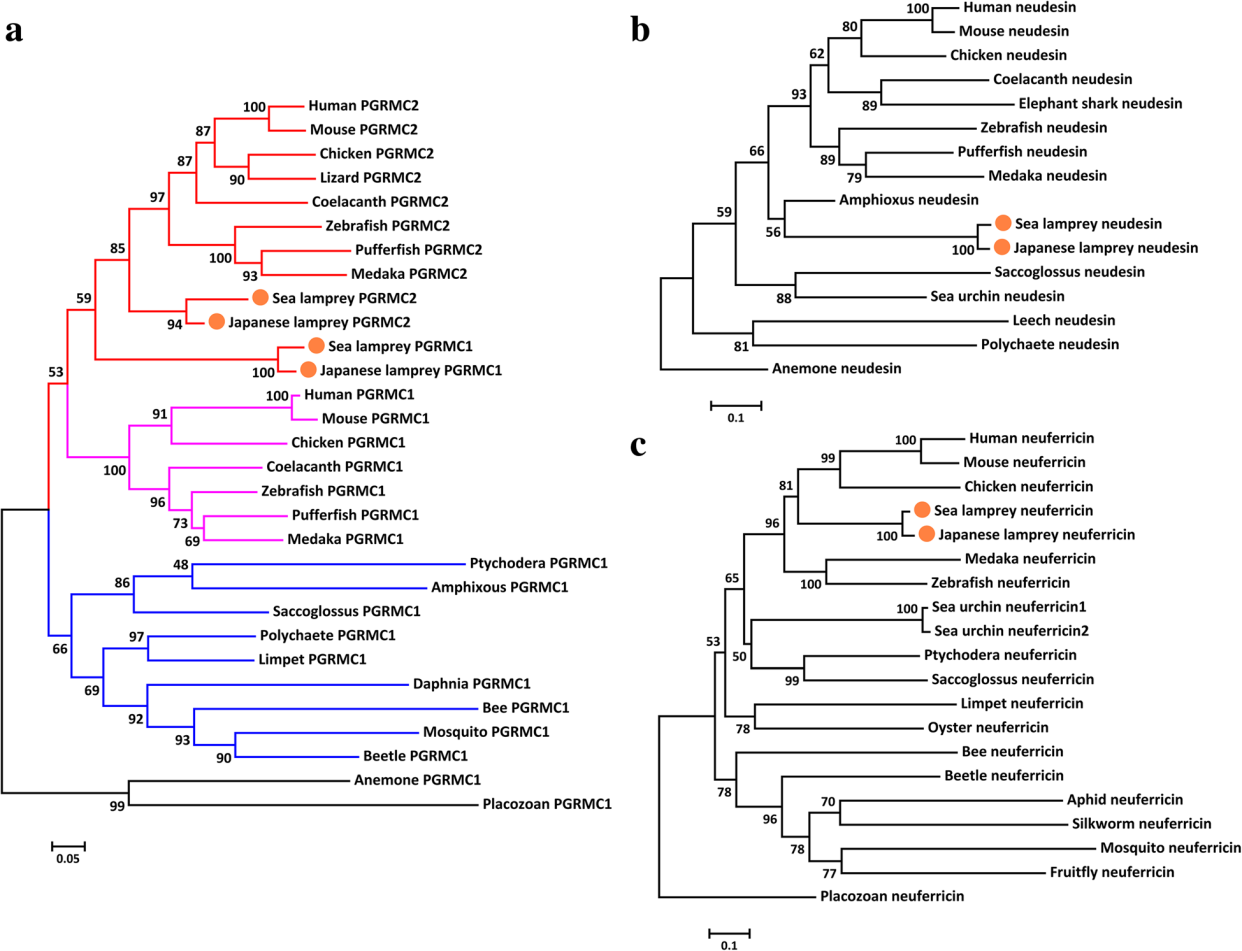
The separated phylogenetic trees of PGRMC1/2 (**a**), neudesin (**b**), neuferricin (**c**) demonstrated the evolutionary relationship among each member of MAPR gene family in metazoans. The trees were constructed using the NJ method with 100 bootstraps. Different clades of PGRMC genes in metazoans are highlighted with different colors (red: vertebrate PGRMC2; pink: vertebrate PGRMC1; blue: invertebrate PGRMC1; black: PGRMC1 out groups). The MAPR genes identified in lampreys are indicated with orange dots

The phylogenetic trees of PGRMC1/2, neudesin and neuferricin were built separately due to their diverse amino acid sequences. In general, the topologies of the phylogenetic trees were consistent with evolutionary relationships among representative species with moderate to high bootstrap values (Fig. 4 and Additional file 10). The phylogenetic tree of PGRMC was clustered into an invertebrate PGRMC1 clade and a vertebrate PGRMC clade. The vertebrate PGRMC clade was further divided into PGRMC1 and PGRMC2 sister groups. However, lamprey PGRMC1 was always clustered with vertebrate PGRMC2 in both NJ and ML trees, probably due to incomplete lamprey PGRMC2 sequences (Fig. 4a and Additional file 10a). The PGRMC1 gene was identified in nearly all metazoan species examined, even in the closest known unicellular living relative of animals, choanoflagellate (*Monosiga brevicollis*) (Table 3 and Additional file 2). On the contrary, the PGRMC2 gene was only identified in vertebrates (Fig. 4a). These results indicated that two copies of PGRMC genes probably derived from duplication of ancestral PGRMC1 sequence and appeared before the speciation of agnathans.

The Neudesin gene was first identified in sea anemone (Cnidaria) and later found as a single copy in invertebrates and vertebrates (Table 3). However, the neudesin gene was not found in several protostomes such as platyhelminthes, nematodes, crustacean and insects (Table 3 and Additional file 2). Neuferricin genes were identified in all metazoan phyla examined except Platyhelminthes (Table 3 and Additional file 2). The lack of neudesin genes in these phyla may be due to incomplete genome assemblies or gene loss.

Protein motif analyses of lamprey MAPR proteins supported the conclusion from phylogenetic analyses that PGRMC2 may have derived from PGRMC1 since both contained acidophilic serine/threonine kinase and tyrosine kinase domains, and PGRMC2 further acquired DNA damage kinase, Erk-D and SH2 domains. Neudesin and neuferricin both contained basophilic kinase and Erk-D domains whereas neuferricin further gained a SH3 and a PDK1 binding domain (Table 2).

### Syntenic analysis of mPR and MAPR genes among five chordates

**Fig. 5:**
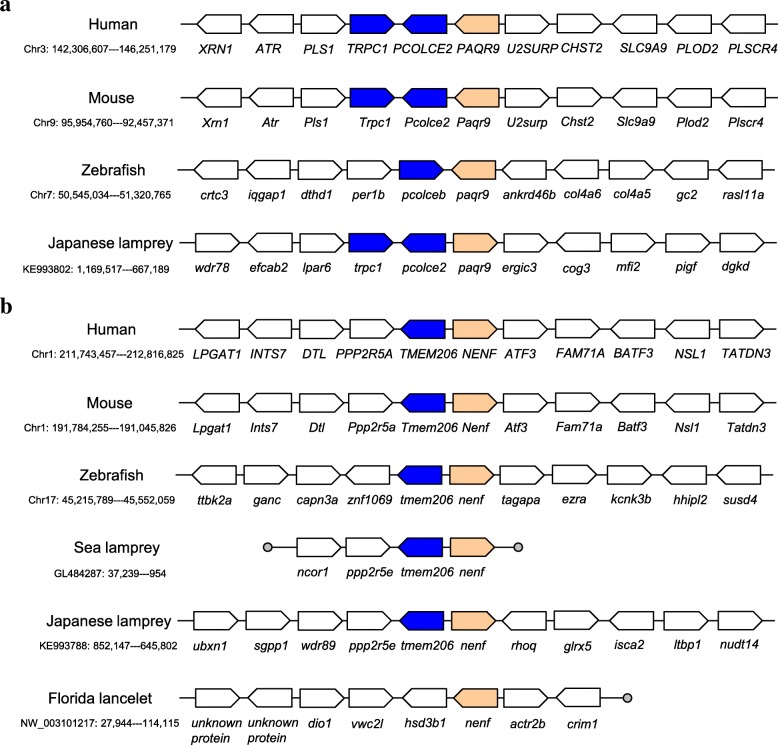
Analysis of conserved synteny blocks harbouring mPRε (**a**) and neudesin (**b**) among five chordates. Solid circle represents the end of scaffold. Orange and blue blocks indicate the PR and its adjacent conserved genes, respectively. Gene names are based on NCBI Map Viewer or blast search against NCBI NR database

Five genes flanking the mPR and MAPR genes were presented and compared among human, mouse, zebrafish, lampreys and Florida lancelet (Additional file 11). In general, gene arrangement was conserved among human, mouse and zebrafish genomes. Lampreys only contained partial gene synteny near mPRε and neudesin, similar to human, mouse and zebrafish. Syntenic genes were not found near mPR and MAPR genes (Fig. 5 and Additional file 11).

The gene arrangement on both sides of PAQR8 (mPRβ) was identical, with nine contiguous genes in human and mouse. Zebrafish genome contained three syntenic genes (Il17a/f3-mcm3) 5′ to paqr8 similar to mouse genome, whereas lampreys and four other species did not exhibit similar gene synteny. In lancelet, we identified nine mPRβ genes. Most of them were in tandem arrangement in two scaffolds, demonstrating gene expansion via tandem duplication (Additional file 11). Compared to the gene arrangement on both sides of PAQR5 (mPRγ) in human, NOX5 gene was translocated to different chromosome in mouse genome. Zebrafish genome contained paqr5a and paqr5b, generated by genome duplication, evidenced from well-conserved synteny 3′ to paqr5a and on both sides of paqr5b, similar to mouse genome. No gene synteny was shared among lampreys and other four species. Compared to gene arrangement on both sides of PAQR6 (mPRδ) in human genome, three tandem copies of Bglap occurred 5′ to Paqr6*,* and the VHLL gene was translocated to a different chromosome in the mouse genome. The zebrafish genome preserved one gene (smg5) 3′ to paqr6, similar to the mouse genome. However, no gene synteny was shared among lampreys and other three species (no mPRδ in lancelet). The gene arrangement on both sides of PAQR9 (mPRε) was identical between human and mouse. Compared to the mouse genome, the zebrafish genome contained one gene (pcolceb) while lamprey preserved two genes (trpc1-pcolce2) 5′ to paqr9 (Fig. 5a).

Compared to gene arrangement flanking PGRMC1 in human genome, KIAA1210 was translocated to different chromosome and Akap17b was inserted at the 3′ end in the mouse genome. Zebrafish, lamprey and lancelet did not share any synteny with human and mouse (Additional file 11). The gene arrangement on both ends of PGRMC2 were identical in human and mouse. Zebrafish shared a syntenic block (larp1b, pgrmc2 and jade1) with an insertion of *asmt2* compared to mouse. The gene arrangement on both sides of NENF (neudesin) was completely identical in human and mouse. Both zebrafish and lamprey (but not lancelet) shared synteny (tmem206 and nenf) with human and mouse (Fig. 5b). Compared to the gene arrangement on both ends of CYB5D2 (neuferricin) in human, RYKP1 gene was translocated to the 3′ end in mouse. Zebrafish and mouse shared a well-conserved syntenic block containing nine contiguous genes including an inversion between p2rx1 and camkk1 (Additional file 11).

## Discussion

### The evolution of nPR, mPR and MAPR genes

Phylogenetic analyses of metazoan PR homologous sequences support the notion that the membrane-bound PRs, mPRs and PGRMC, likely evolved before nPR since they appeared at the basal animal lineage, and probably originated from an ancestral Hly-III-containing protein and cyt-b5-containing protein, respectively [13]. During metazoan evolution, new mPR and MAPR sequences probably derived from gene duplications. The genes for nPR appeared later in the vertebrate lineage, tracing back to an early common ancestor with ER [47], and a last common ancestor with corticoid receptor [29]. The appearance of nPR in vertebrates endowed a new mode of P_4_ function by directly acting on the genome to regulate gene transcriptions, in addition to the cellular responses through the membrane receptors.

### The conservation and expansion of mPR gene family during metazoan evolution

**Fig. 6:**
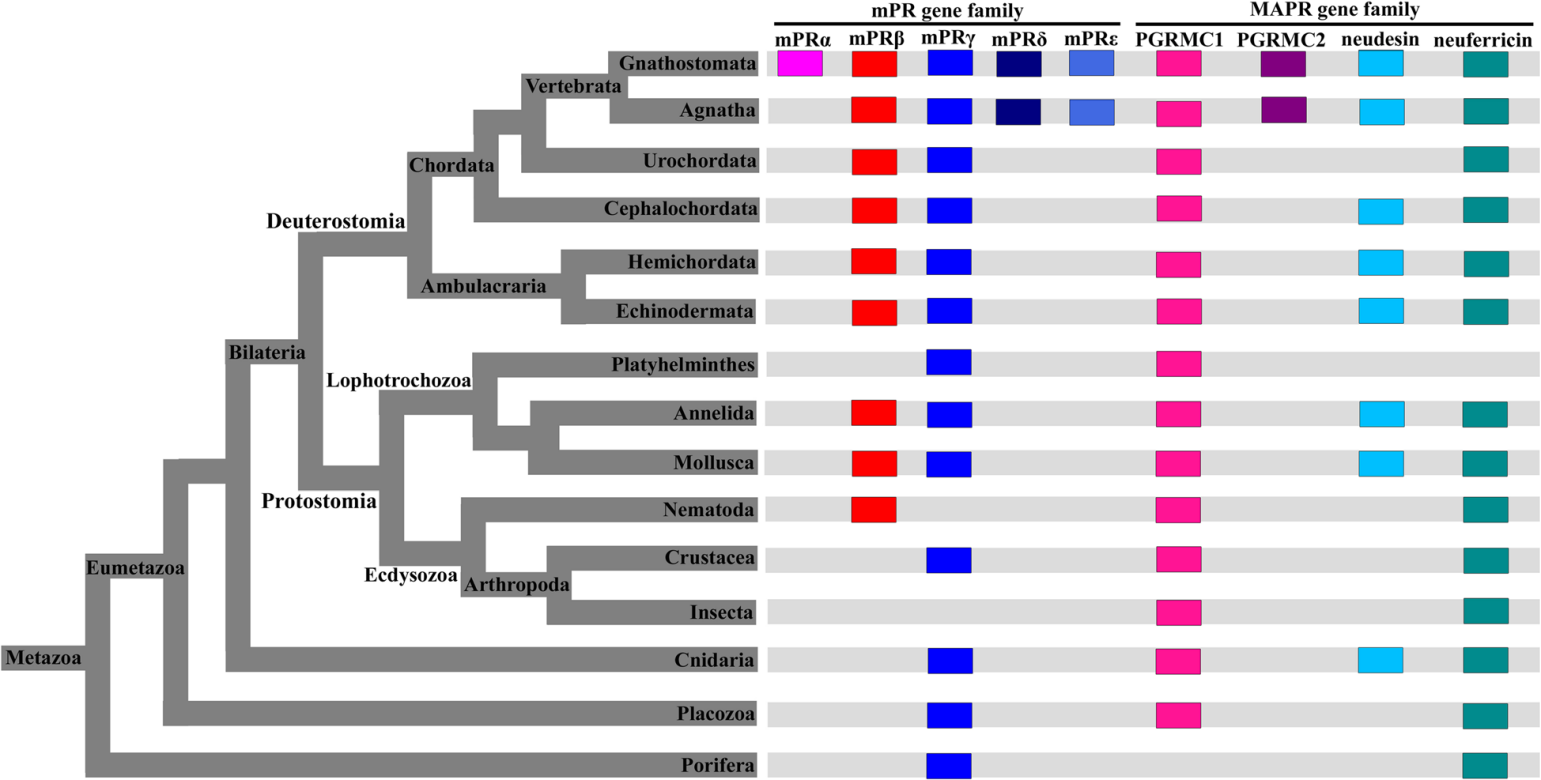
Members of mPR and MAPR gene families in different taxonomic units of Metazoa. A schematic phylogenetic tree showed the relationships among major taxonomic units of Metazoa. The mPR and MAPR members presented in a taxonomic unit is indicated in colored boxes

The mPR gene family is conserved in all metazoan phyla examined, and its ancestral sequences can be traced back to Eubacteria [48]. The mPR gene family expanded from one (mPRγ) to five members (mPRα, β, γ, δ, and ε) during metazoan evolution (Fig. 6). Our phylogenetic analyses revealed that the mPRγ gene first appeared in non-bilaterians and the mPRβ gene arose from the early duplication of the mPRγ gene in bilaterians. mPRδ and ε genes also arose from duplications of the mPRγ gene, but these events occurred much later in vertebrate lineages from lamprey to human. On the contrary, the mPRα gene first appeared in cartilaginous fishes, probably from the duplication of the mPRβ gene after the agnathan-gnathostome divergence (Fig. 6). In addition, mPRβ and γ genes underwent species-specific duplications and expanded into multiple copies (> 2) in several species such as Florida lancelet, owl limpet, pacific oyster, sea urchin, placozoan and sea anemone (Additional file 2). In Florida lancelet and sea anemone, the copy number for mPRβ and mPRγ genes reached up to nine and seven, respectively (Table 3). The multiple copies in species-specific duplications were often in tandem arrangement via tandem duplication as demonstrated in lancelet mPRβ (Additional file 11). Only mPRα showed lineage-specific duplication in teleosts, probably due to teleost-specific third-round whole genome duplication (3R-WGD). Unlike mPRα, β or γ, neither mPRδ nor ε showed lineage- or species-specific duplication and contained only one copy (Table 3).

Surprisingly, none of the mPR members were identified in all six insect genomes examined (Fig. 6 and Additional file 2). Further investigation of additional insect genomes is required to conclude whether the mPR gene family is indeed lost in the insect lineage.

### Conservation of the MAPR gene family during metazoan evolution

MAPRs are a group of rather small, partially homologous proteins, which share a similar non-covalent heme-binding domain related to cytochrome b5 [49]. The heme-binding cyt-b5 domain may serve as a template for more recently-evolved novel ligand binding pockets, such as a steroid-binding site in the MAPR proteins [12]. Most proteins with a cyt-b5 domain are linked to cell membranes, either directly or by forming part of the membrane-associated complexes [12]. The proximity of cell membranes to a heme-binding template pocket allowed the binding specificity for membrane-soluble molecules such as steroids. Thus, MAPRs may represent an adaptation to use steroids as triggers for fast-acting mechanisms [12]. Progesterone-binding activity has rarely been demonstrated directly in purified MAPR proteins [24]. Recent studies showed that progesterone binds to human PGRMC1 [24], similar to the first PGRMC1 isolated from porcine liver cell membranes with high P_4_-binding affinity [23]. Another study also demonstrated that human PGRMC1 acted as an adaptor protein for the mPR protein complex [25]. Therefore, PGRMC1 is likely involved in progesterone signaling pathway as either a receptor or an adaptor protein, or both.

The MAPR genes likely evolved in plants, fungi and metazoans and PGRMC1-like genes apparently evolved in early eukaryotes [3, 12]. Ancestral metazoans may contain three MAPR genes, corresponding to ancestral PGRMC, neudesin, and neuferricin [3]. A previous study showed that PGRMC1 and PGRMC2 diverged in early chordates, or possibly in insect lineages [3]. Our phylogenetic analyses demonstrated that the MAPR gene family initially contained three members (PGRMC1, neudesin, and neuferricin), supporting the notion that PGRMC1 and PGRMC2 diverged in vertebrate lineage since two PGRMC members first appeared in lamprey genomes (The PGRMC genes of fruit fly identified in [3] were in fact three copies of PGRMC1, not PGRMC2). The choanoflagellate, the closest unicellular living relative of the metazoans, contained only PGRMC1 and neuferricin (Table 3). In addition, MAPR members showed some species-specific duplication, resulting in three copies of PGRMC1 in polychaete worm and fruit fly, and two copies of neuferricin in sea anemone, sea urchin and lancelet, which may derive from tandem duplication (Additional file 11). Vertebrate species harbored all four MAPR members without obvious expansions from lamprey to human, even in teleosts with 3R-WGD.

### Progesterone signaling: from membrane to nucleus

It appears that the mPR or MAPR genes evolved earlier than the nPR genes [29–35]. It is believed that nPR genes descended from a single promiscuous ancestral steroid receptor gene in protostomes, which branched off from the rest of the nuclear receptor superfamily early in animal evolution [39]. The specificity in more recent receptor proteins has been tuned by natural selection [50]. The ability of ancient receptors to interact with many ligands allowed species with small protein repertoires to carry out broad biological activities and promoted future evolution of new functions. Specialization in more recent receptor proteins provides greater efficiency, finer regulation, or prevention of deleterious interactions [50]. The ligand binding ability likely evolved through a process of molecular exploitation; i.e., the evolution of new ligand/receptor interactions when an older receptor is co-opted to bind a newly-evolved ligand [39, 50]. Indeed, based on shared steroid metabolic pathways among different taxa, Markov et al. deduced that the estrogen-binding ability evolved before the ability to bind other side-chain cleaved steroids and the ancestral ligand to the first steroid-binding receptor was not an 18-carbon estrogen but likely a molecule with a side chain in addition to the aromatic A-ring specific to vertebrate estrogens [51].

P_4_-sensitivity of these receptors in early metazoa probably acted as a sensor for exogenous progestins produced by other taxa in the environment [39, 52]. The *cyp11a* gene, encoding the P450 enzyme that converts cholesterol to pregnenolone, exists in deuterostomes [53]. The genes encoding 3β-HSD enzymes that convert pregnenolone to P_4_ are present in the fungus *Aspergillus fumigatus* [54]. P_4_ and its synthetic enzymes have been unambiguously detected in cephalochordates [55], and P_4_ has been identified in mollusks by gas or liquid chromatography/mass spectrometry [56, 57]. High levels of exogenous P_4_ inhibit growth in fungal yeast cells via membrane P_4_ receptors [32]. Experimental evidence also demonstrates that functional progesterone membrane-associated receptor exists in invertebrate rotifer *Brachionus manjavacas* [33]. Therefore, the P_4_ binding ability of mPR, MAPR and nPRs likely evolved through molecular exploitation [39, 50], and the function of these receptors in early metazoa is probably not for reproduction, but rather for chemoreception [39, 52, 58, 59].

Most membrane receptor families evolved at the advent of multicellularity and coincided with the need for coordinated cellular behaviors [60]. The mPRs containing 7TM domains were thought to be unique GPCRs [19, 61]. GPCRs are among the first group of receptors to emerge in unicellular organisms [62, 63]. Their ligands, including light-sensitive compounds, odorants, hormones, and neurotransmitters, induce a conformational change upon binding with the receptors, which activates two principal signal transduction pathways, the cAMP and the phosphatidylinositol signaling pathways, resulting in signal amplification from the exterior to the interior of cells [64, 65].

## Conclusions

In conclusion, non-classical mPR and MAPR genes first evolved in non-bilaterians and classical nPR genes evolved later in basal vertebrates. Sequence repertoires for mPRs and MAPRs in vertebrates likely originated from an ancestral metazoan sequence and expanded via several gene duplication events.

## Methods

### Identification of nPR, mPR and MAPR sequences in lampreys

The genomic resources used for these analyses include the nucleotide and protein sequences from the sea lamprey and Japanese lamprey genomes, and a sea lamprey transcriptomic sequences. The sea lamprey transcriptomic sequences were obtained through assembling 86 Illumina RNA-Seq samples from various tissues across different developmental stages. The RNA-Seq data were generated during the sequencing project of sea lamprey genome. Sixteen samples have been published in [43] and the remaining data has not been published. The Lamprey tissues for Illumina transcriptomic assembly include 1) four development stage embryos, 2) larval gill, kidney, liver and intestine, 3) seven metamorphosis stage liver, intestine and kidney, 4) juvenile and adult intestine, liver and kidney, 5) other adult tissues (see more information in [43, 66]). All available nPR, mPR and MAPR sequences of human (*Homo sapiens*), mouse (*Mus musculus*) and zebrafish (*Danio rerio*) were retrieved from Ensembl (http://www.ensembl.org/index.html) and used as queries to search against the genomic resources by stand-alone BLASTP or TBLASTN. The coding regions from retrieved RNA-Seq transcripts were predicted using GETORF of the EMBOSS online tool (EMBOSS GUI v1.14). The resulting protein sequences were validated by BLASTP against NCBI non-redundant protein sequence database (nr) to ascertain that these identified proteins are actual progesterone receptors and not some members of Class II PAQR family. These sea lamprey and Japanese lamprey PR sequences have been deposited in GenBank and listed in Additional file 1. The conserved protein domains were predicted with SMART (simple modular architecture research tool) [67]. The transcripts of the identified genes were mapped to lamprey genomic sequences for gene structure analyses and then drawn with FancyGene [68].

### Identification of nPR, mPR and MAPR homologous sequences in selective metazoans

Deduced amino acid sequences from different metazoan taxonomies were downloaded from the Ensembl genome browser (release75), including Porifera (*Amphimedon queenslandica*), Placozoa (*Trichoplax adhaerens*), Cnidaria (*Nematostella vectensis*), Insecta (*Acyrthosiphon pisum*, *Anopheles gambiae*, *Apis mellifera*, *Bombyx mori*, *Drosophila melanogaster*, and *Tribolium castaneum*), Crustacea (*Daphnia pulex*), Nematoda (*Caenorhabditis elegans*), Mollusca (*Crassostrea gigas* and *Lottia gigantea*), Annelida (*Capitella teleta* and *Helobdella robusta*), Platyhelminthes (*Schistosoma mansoni*), Echinodermata (*Strongylocentrotus purpuratus*), Hemichordata (*Saccoglossus kowalevskii* and *Ptychodera flava*), Urochordata (*Ciona intestinalis* and *C. savignyi*), Chondrichthyes (elephant shark, *Callorhinchus milii*), Osteichthyes [(spotted gar, *Lepisosteus oculatus*), takifugu (*Takifugu rubripes*), medaka (*Oryzias latipes*), and coelacanth (*Latimeria chalumnae*)], Amphibia (*Xenopus tropicalis*), Reptilia (*Anolis carolinensis*) and Aves (*Gallus gallus*). Amino acid sequences of Cephalochordata (*Branchiostoma floridae*) were downloaded from NCBI RefSeq (http://www.ncbi.nlm.nih.gov/refseq/). These amino acid sequences were subjected to BLASTP search against the nPR, mPR and MAPR proteins from human, mouse and zebrafish genomes. The BLAST hit proteins were then confirmed by BLASTP against NCBI nr database to ascertain that these identified proteins are actual progesterone receptors and not some members of Class II PAQR family.

### Phylogenetic analyses of nPR, mPR and MAPR homologous sequences

Representatives of nPR, mPR and MAPR protein sequences identified from aforementioned metazoan species were used for phylogenetic analyses (Additional file 2). In addition, ER and estrogen-related receptor (ERR) gene sequences were chosen as the out groups to infer the origin and evolution of nPR in metazoans (Additional file 2), including sequences from human, mouse, zebrafish, lampreys, Florida lancelet (*B. floridae*), fruit fly, red flour beetle (*T. castaneum*) and placozoa (*T. adhaerens*). Protein sequences in each gene family were aligned by ClustalW2 [69].

Phylogenetic analyses were performed using both neighbor joining (NJ) and maximum likelihood (ML) approaches with 100 bootstrap replicates using MEGA6 software package [70]. The NJ and ML trees for selected PR proteins were built with a JTT substitution model. In the NJ trees, gaps/missing data were treated by paired deletion, whereas in the ML trees, all sites were used.

### Syntenic comparisons of mPR and MAPR genes among five chordates

Protein-coding genes adjacent to mPR and MAPR genes in human, mouse, zebrafish and Florida lancelet genomes were identified from NCBI Map Viewer. The gene arrangements in lampreys were investigated with lamprey genome annotation files. The gene names of lamprey and Florida lancelet were further confirmed by BLASTP against NCBI human, mouse, zebrafish and nr databases. Five genes adjacent to mPR and MAPR genes in both directions (5′ and 3′) were presented and compared among human, mouse, zebrafish, lampreys and Florida lancelet.

### Protein motif analyses

Protein motifs were predicted using the Scansite cell signaling interactions prediction “MotifScan” module (https://scansite4.mit.edu/4.0/#scanProtein) under medium and high stringency settings.

## Additional files

Additional file 1: Sequences of nPR, mPR and MAPR genes identified in Japanese lamprey. (TXT 11 kb)

Additional file 2: Species selected for phylogenetic analyses of nPR, mPR and MAPR genes. (XLSX 20 kb)

Additional file 3: Gene structure of nPR, mPR and MAPR genes in human, mouse and zebrafish. (PDF 368 kb)

Additional file 4: Predicted motif sequences for lamprey PRs using the Scansite cell signaling interactions prediction “MotifScan” module. (XLSX 15 kb)

Additional file 5: A phylogenetic tree constructed by the ML method demonstrates the evolutionary relationship among nPR, ER and ERR in metazoans. (PDF 12 kb)

Additional file 6: Gene location of mPR and MAPR genes in sea lamprey and Japanese lamprey. (XLSX 30 kb)

Additional file 7: Gene structure of four transcript isoforms of sea lamprey mPRβ. (JPG 1194 kb)

Additional file 8: Gene structure of four transcript isoforms of sea lamprey mPRδ. (JPG 1112 kb)

Additional file 9: A phylogenetic tree constructed by the ML method demonstrates the evolutionary relationship among five members of mPR gene family in metazoans. (PDF 16 kb)

Additional file 10: Phylogenetic trees constructed by the ML method demonstrate the evolutionary relationship of PGRMC, neudesin and neuferricin in metazoans. (JPG 4300 kb)

Additional file 11: Syntenic analysis of mPRs and MAPRs genomic sequences among human, mouse, zebrafish and lampreys. (PPTX 169 kb)

## Data Availability

All data analyzed in this study are included within the article and its additional files. The PR gene sequences of sea lamprey have been deposited in GenBank database (accession numbers: KT970648-KT970662) whereas the Japanese lamprey PR gene sequences are included in Additional file 1.

## Abbreviations

3R-WGD: Third-round whole genome duplication
7TM: Seven trans-membrane
CDS: Coding DNA sequence
cyt-b5: Cytochrome b5
DBD: DNA-binding domain
ER: Estrogen receptor
ERR: Estrogen-related receptor
GPCR: G-protein coupled receptor
LBD: Ligand-binding domain
MAPR: Membrane-associated progesterone receptor
mPR: Membrane progestin receptor
nPR: Nuclear progesterone receptor
P_4_: Progesterone
PAQR: Progestin and adipoQ receptor
PGRMC1: Progesterone receptor membrane component 1
PR: Progesterone receptor
UTR: Untranslated region
