# Supplementary figures and images for: Genomic sequence analyses of classical and non-classical lamprey progesterone receptor genes and the inference of homologous gene evolution in metazoans
Source: BMC Evol Biol. 2019 Jul 1;19:136. doi: 10.1186/s12862-019-1463-7 (PMC6604198; doi:10.1186/s12862-019-1463-7)

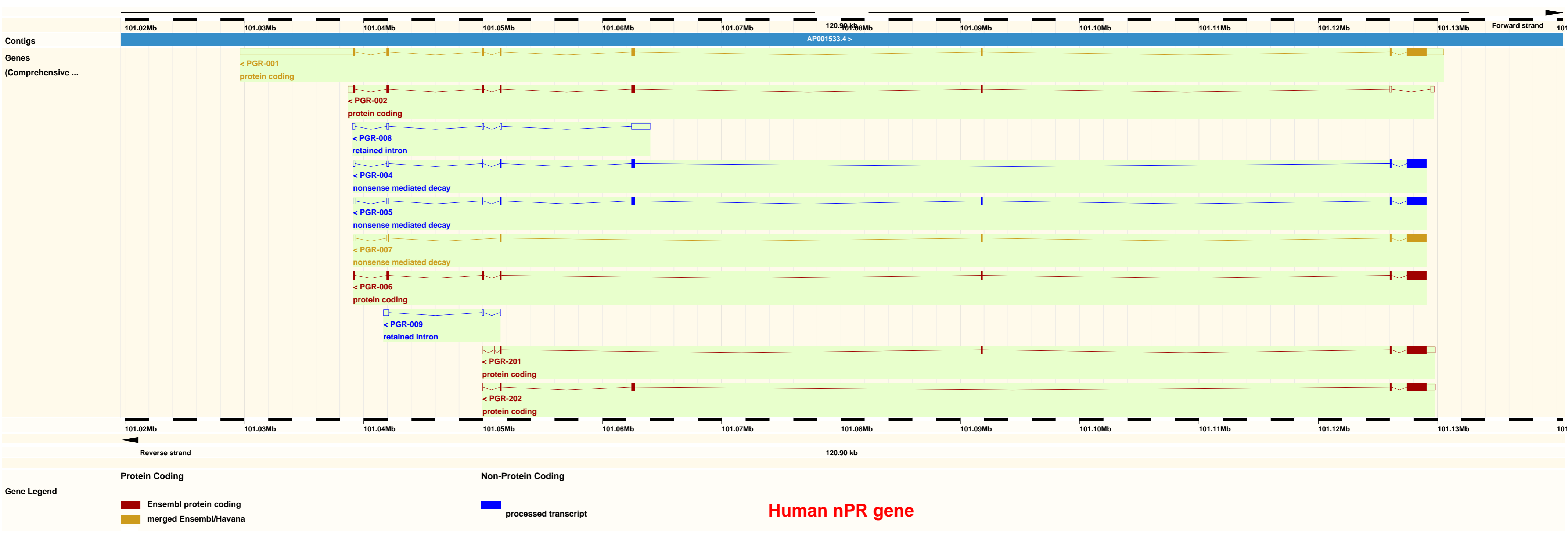

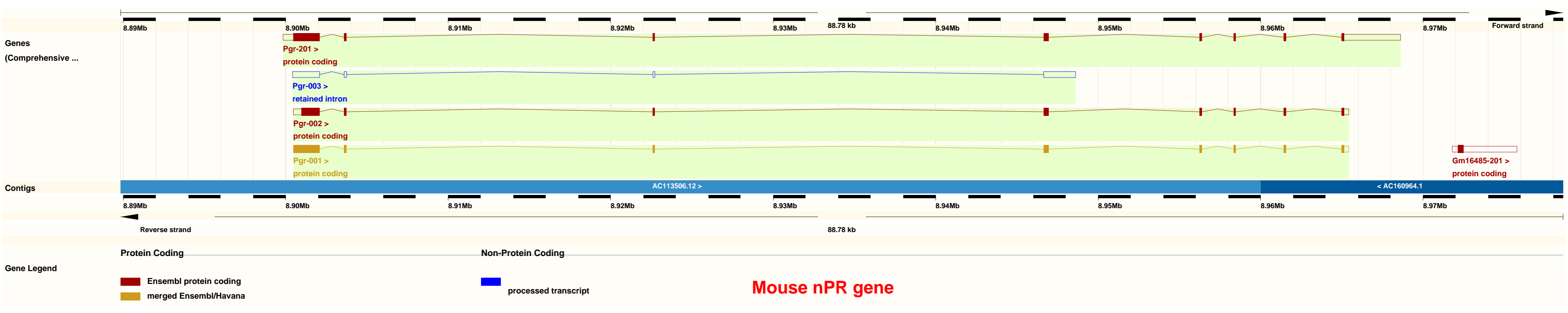

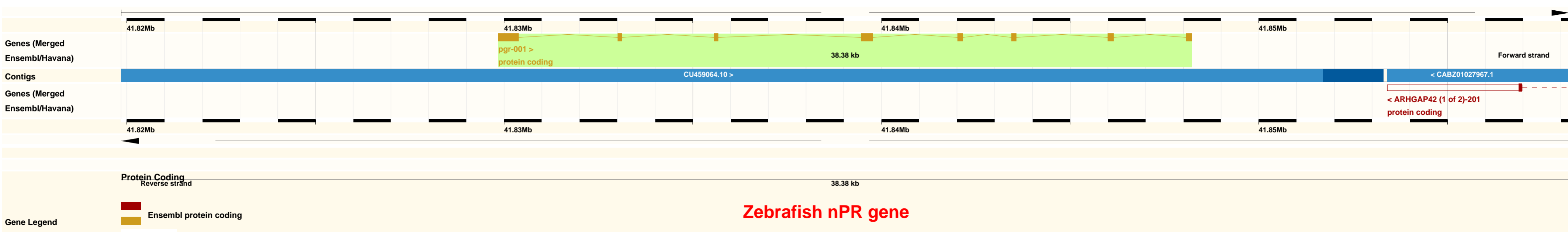

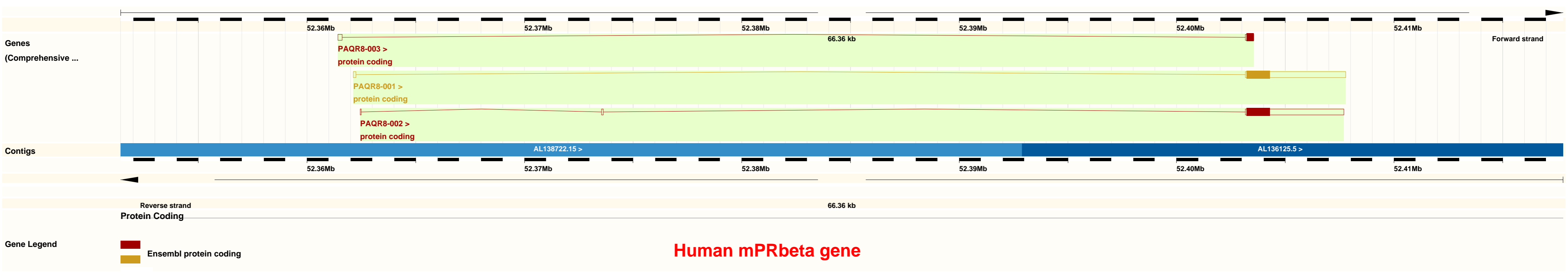

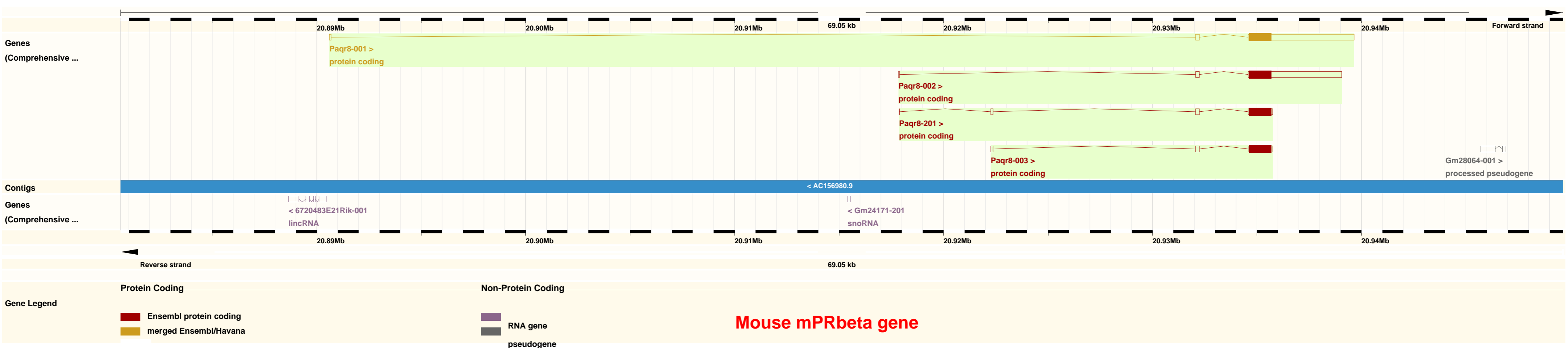

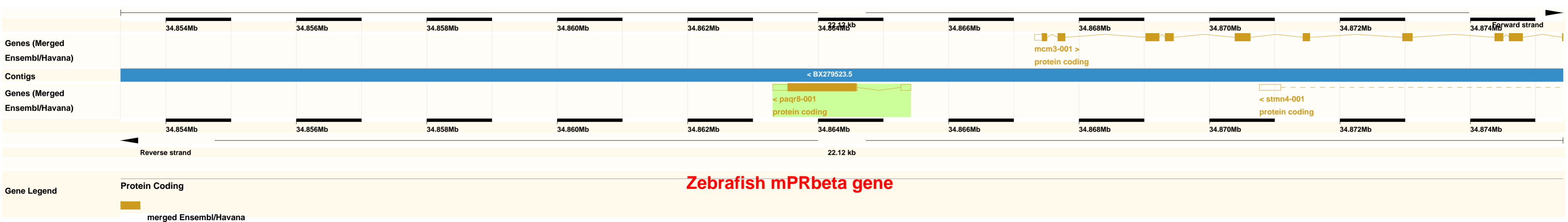

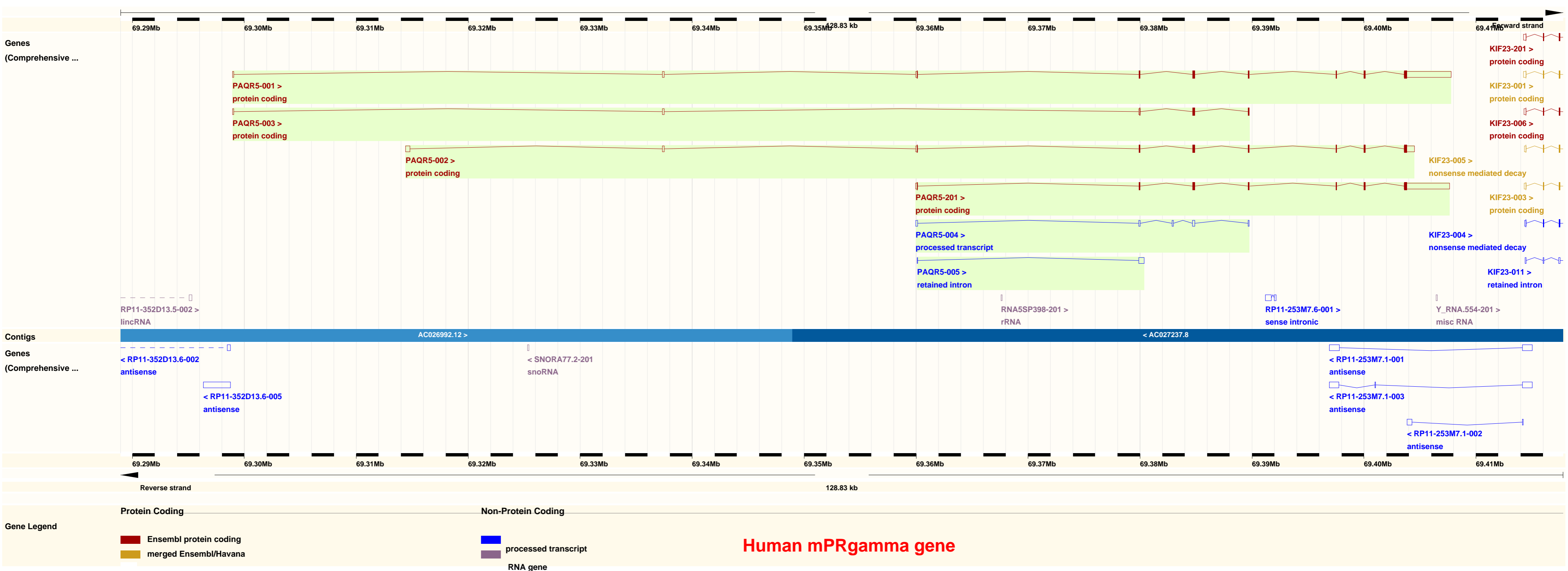

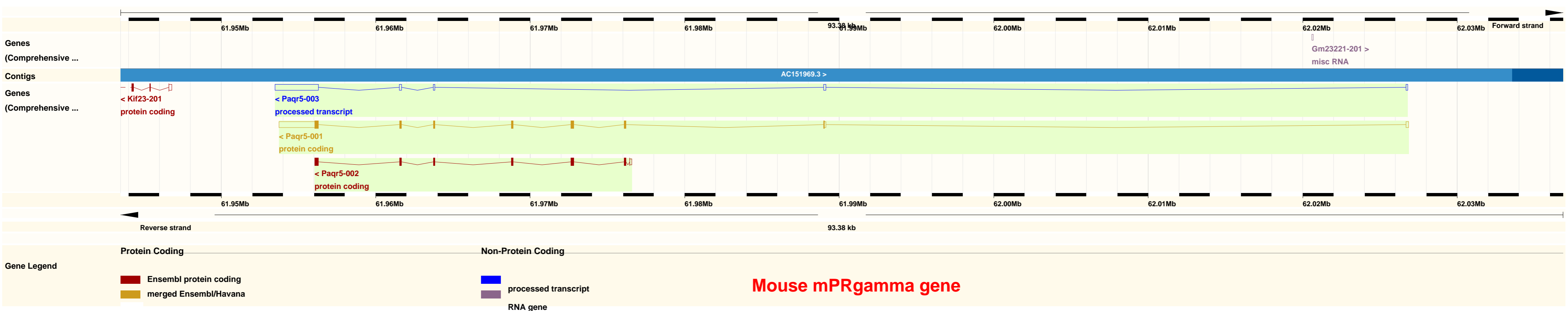

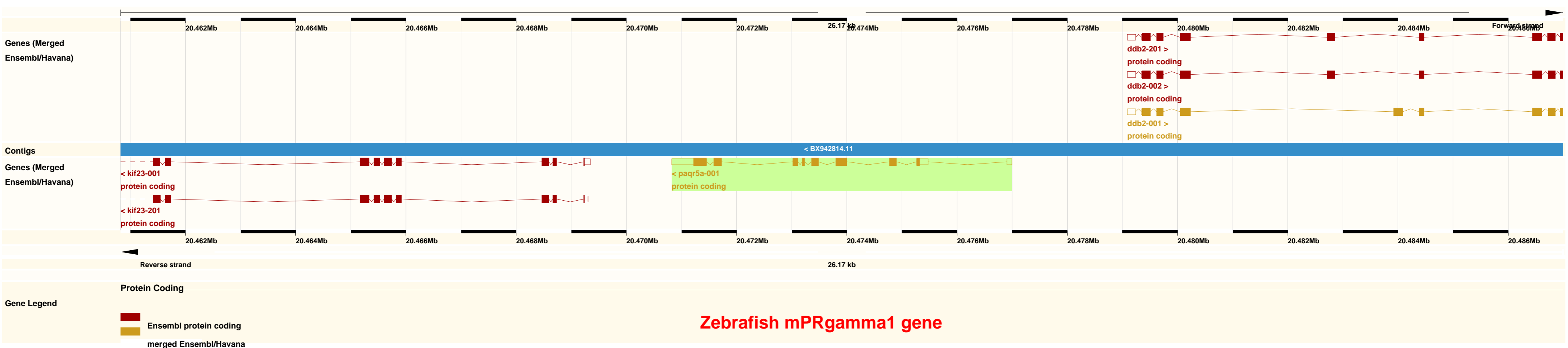

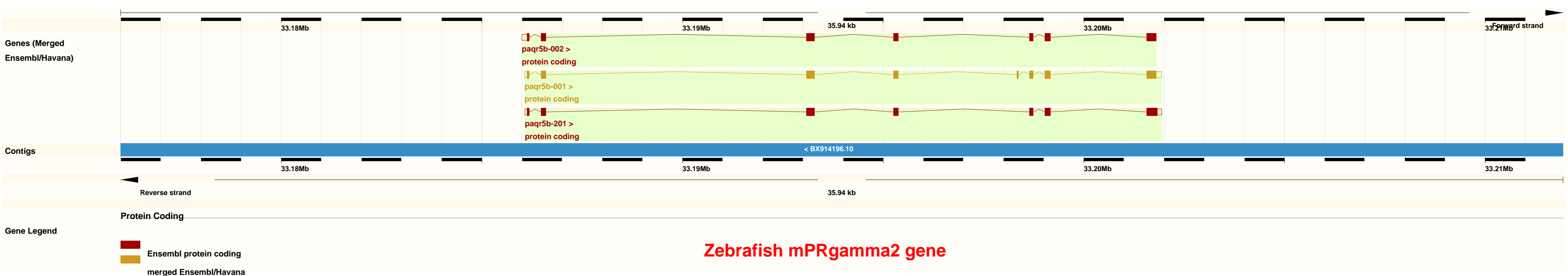

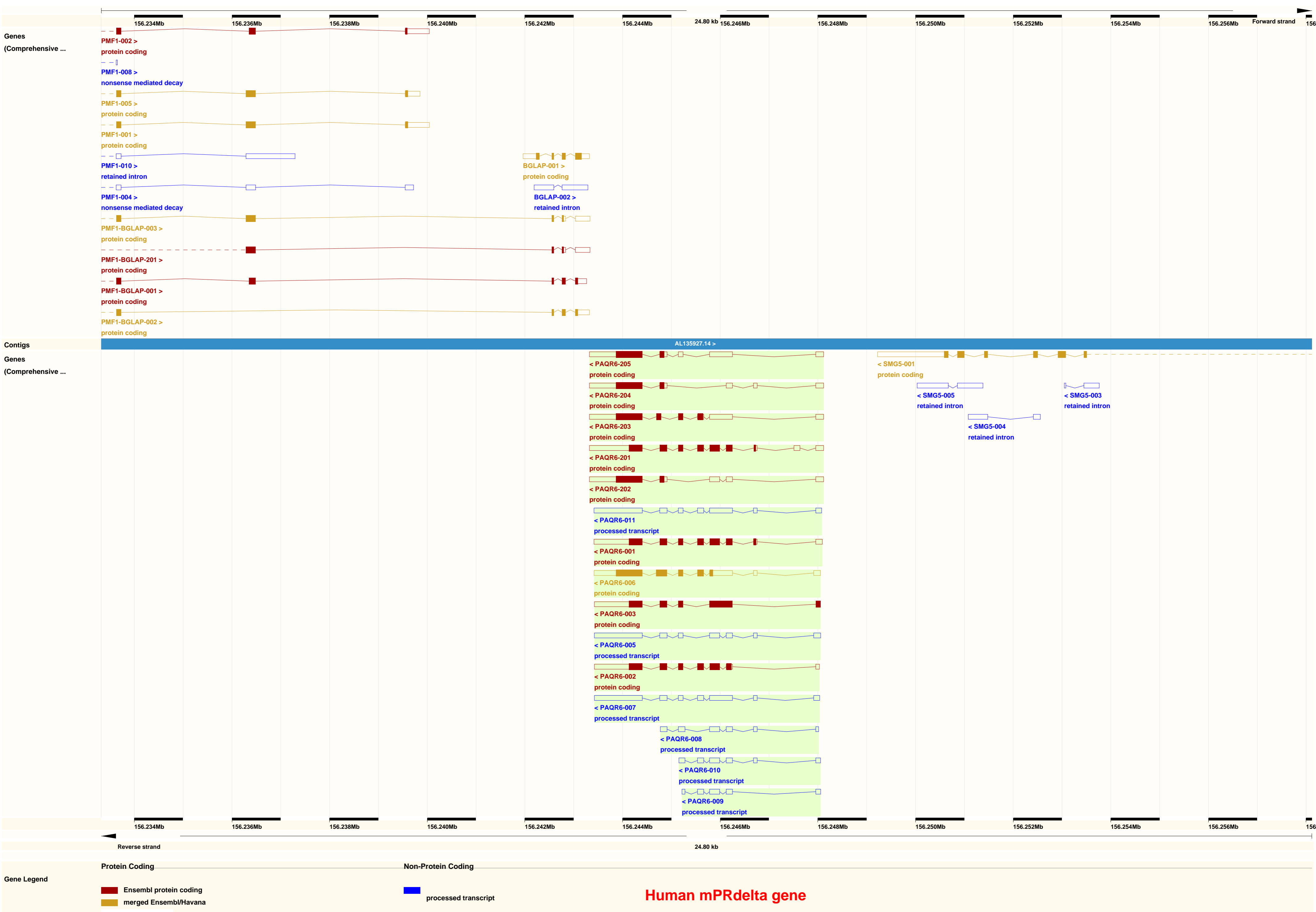

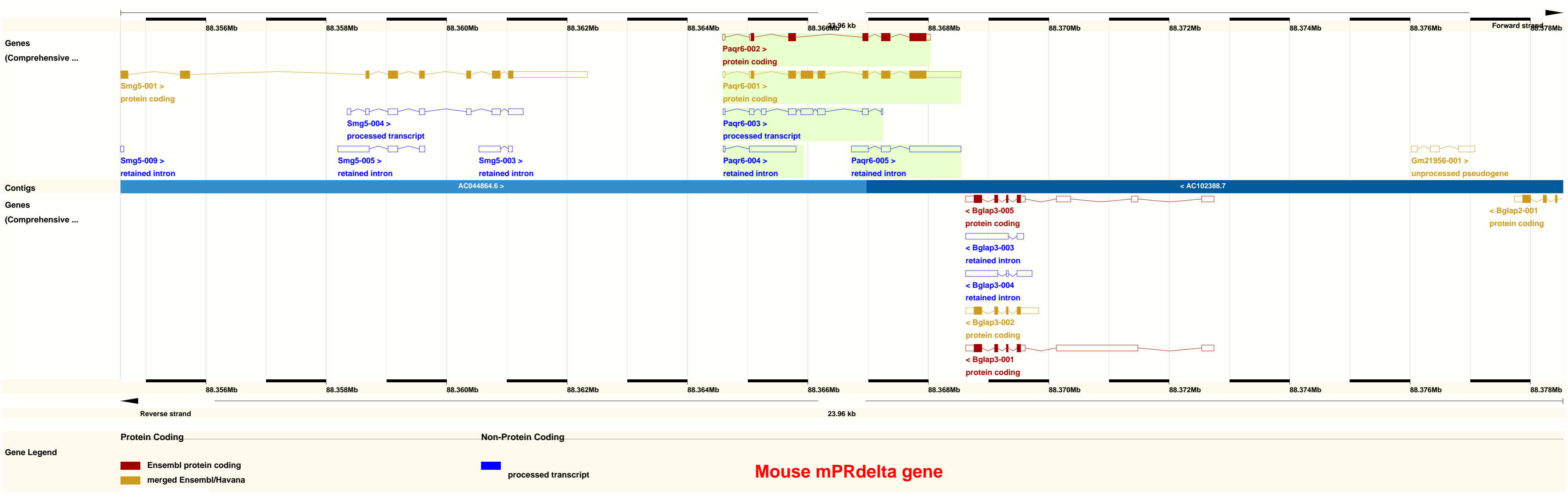

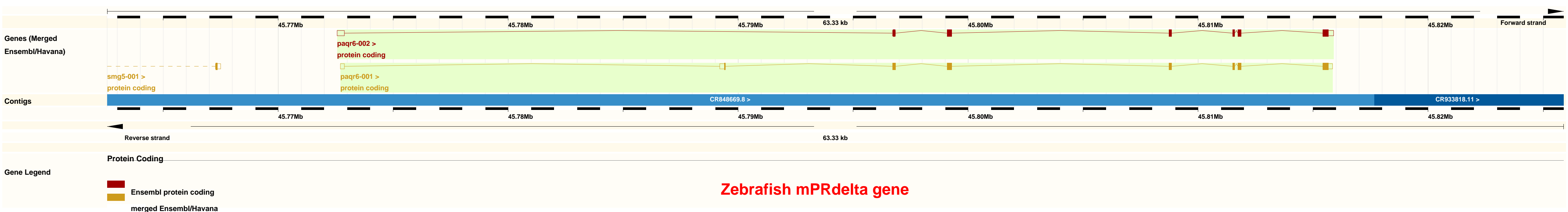

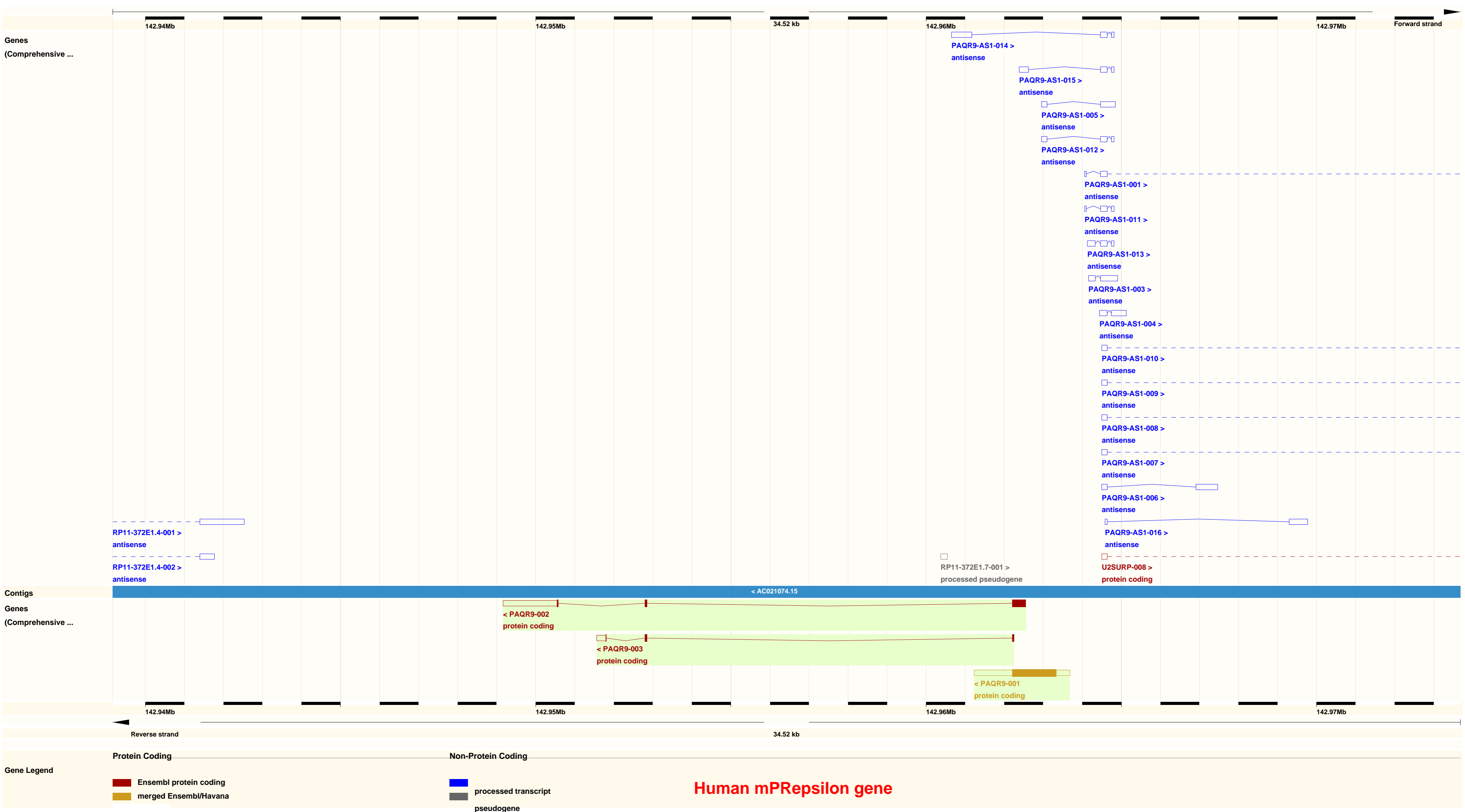

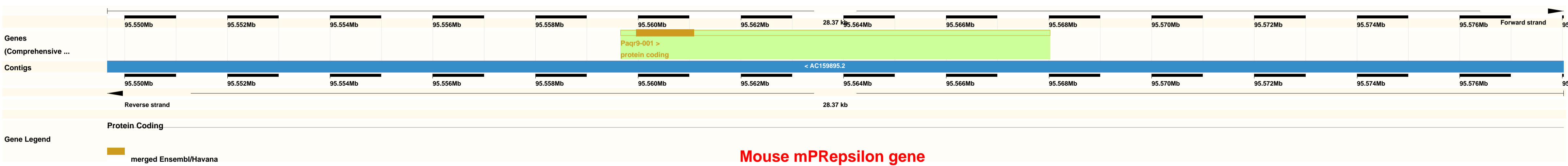

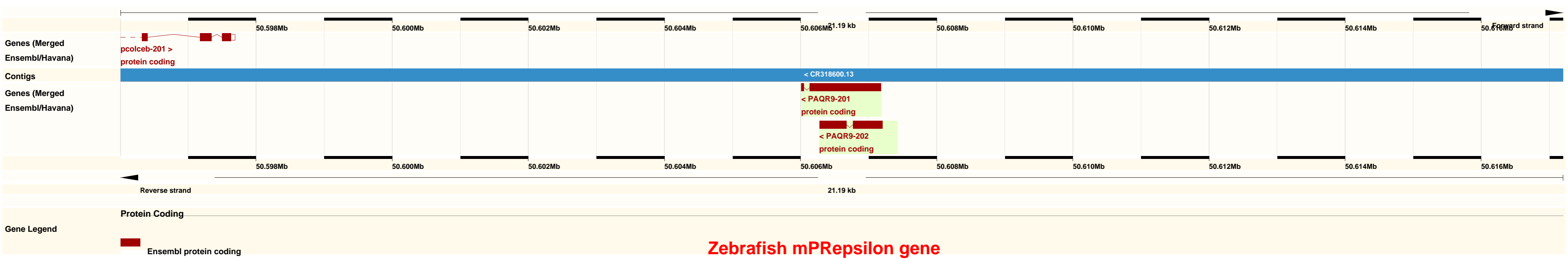

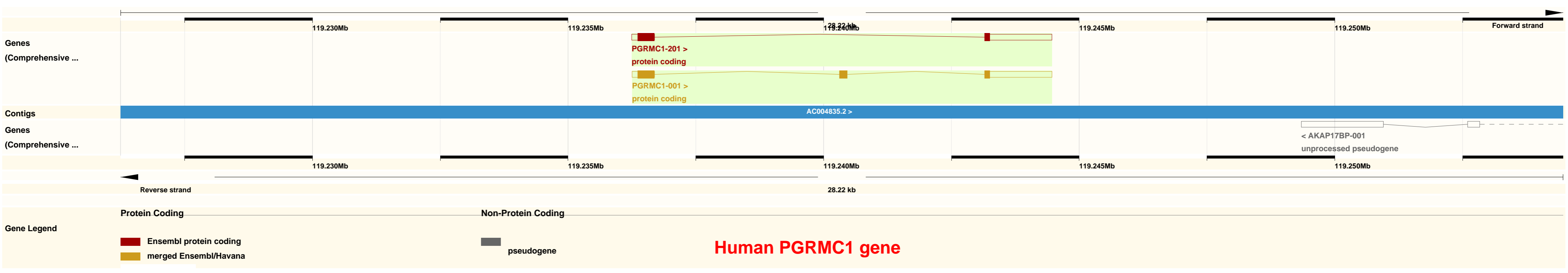

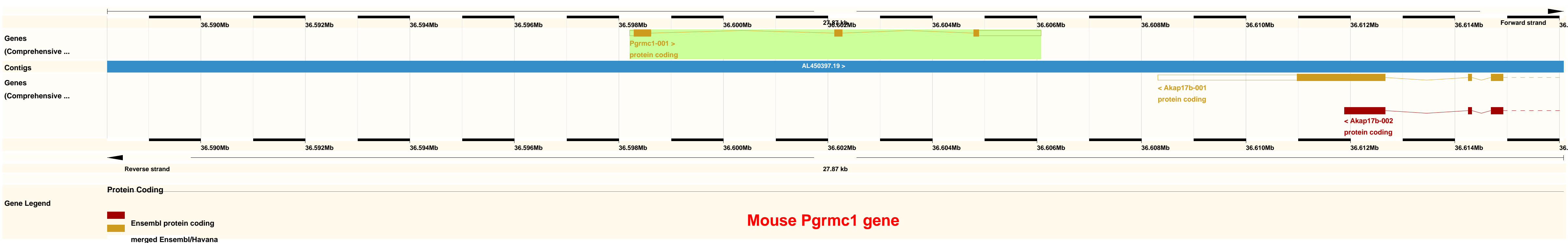

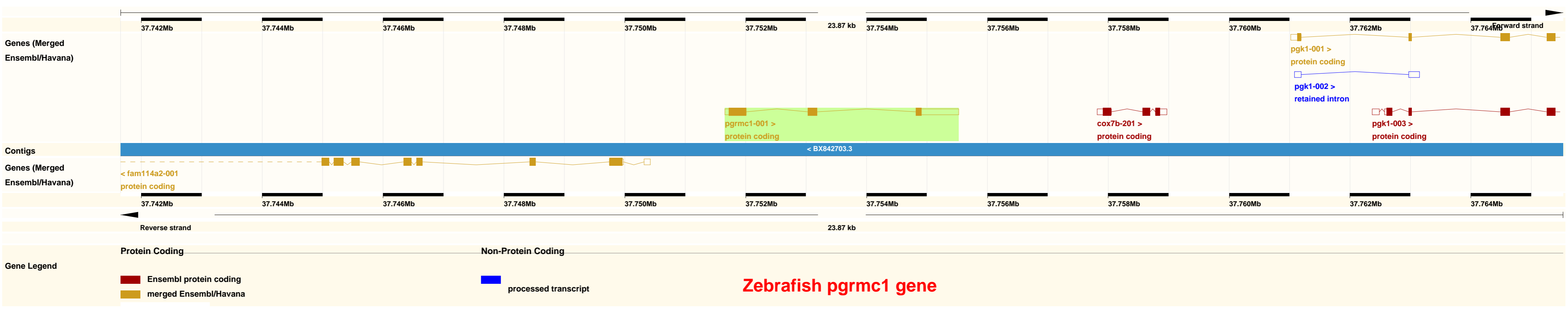

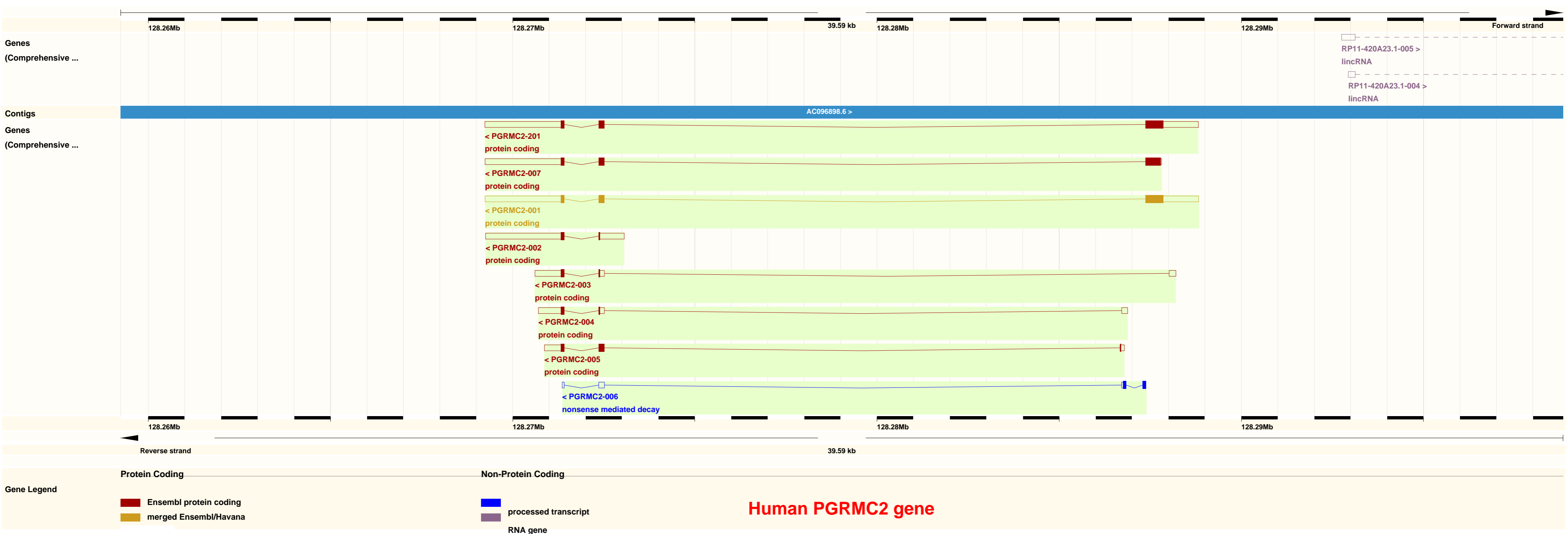

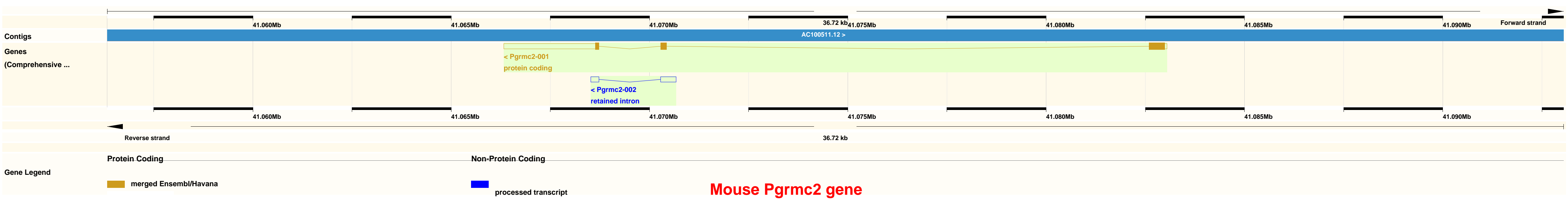

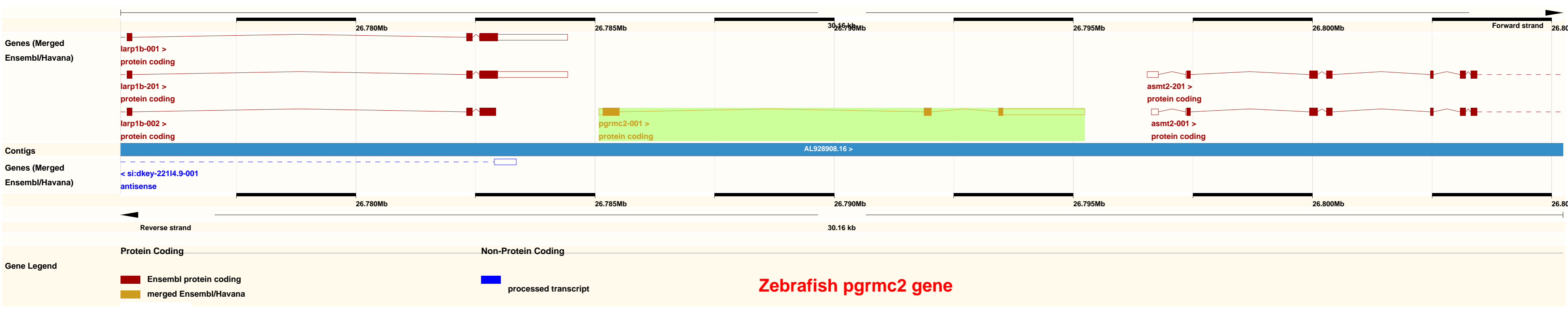

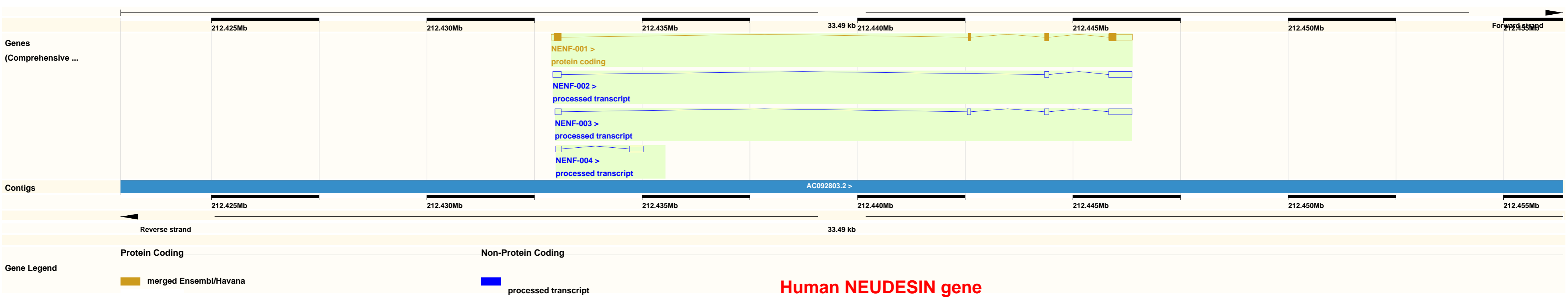

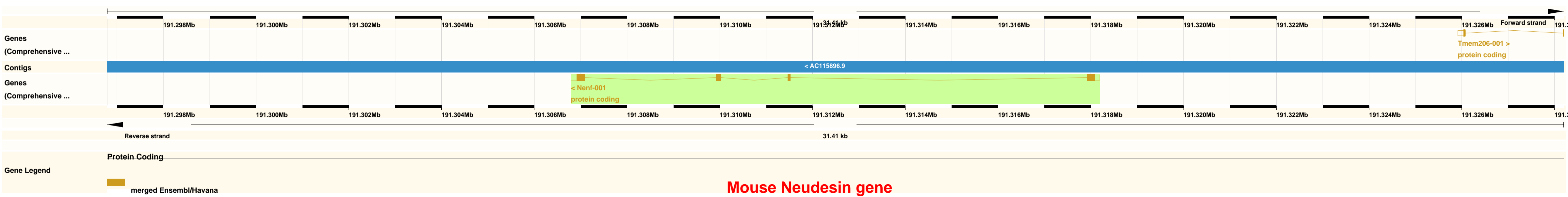

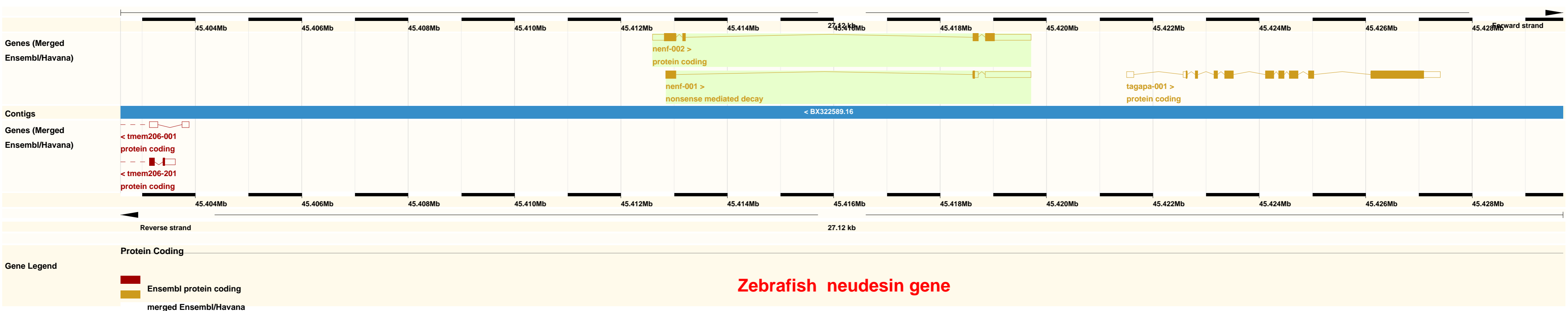

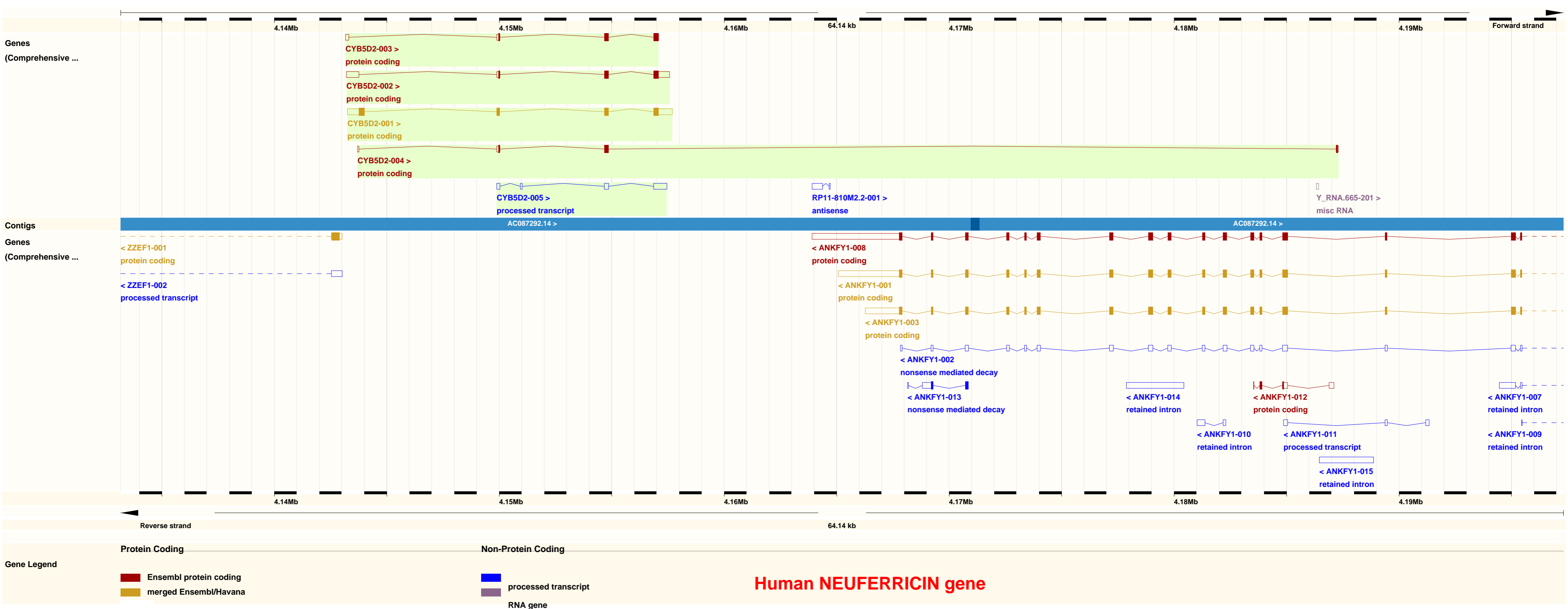

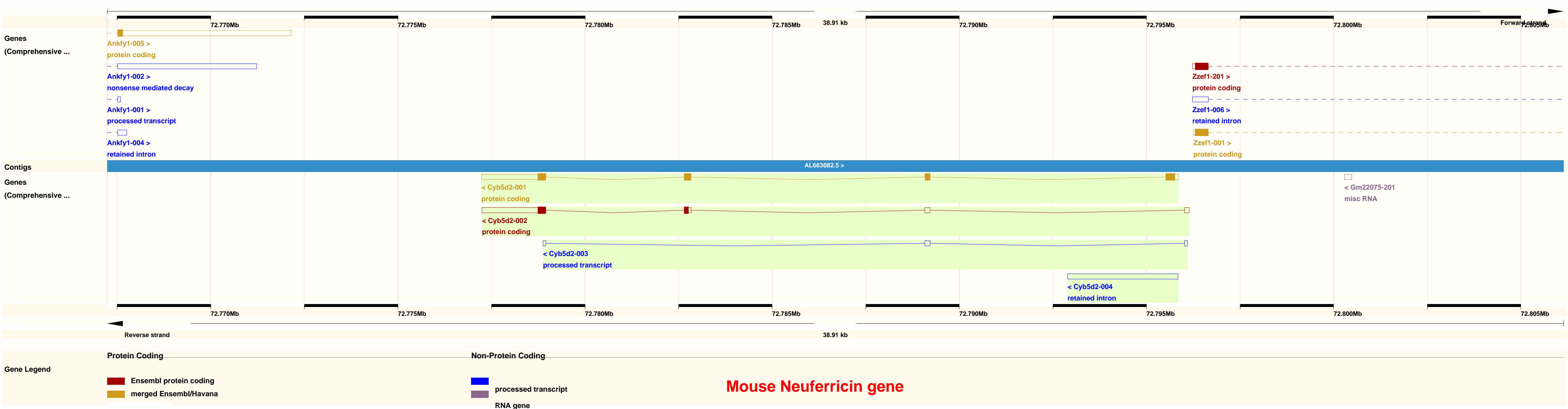

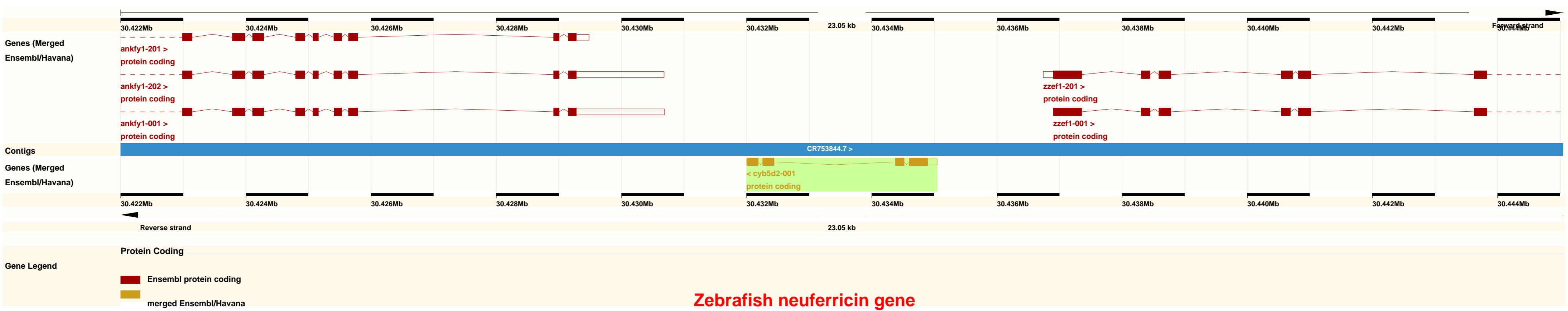

Supplement: Supplementary file 3 — Gene structure of nPR, mPR and MAPR genes in human, mouse and zebrafish. (PDF 368 kb) [file 12862_2019_1463_MOESM3_ESM.pdf]

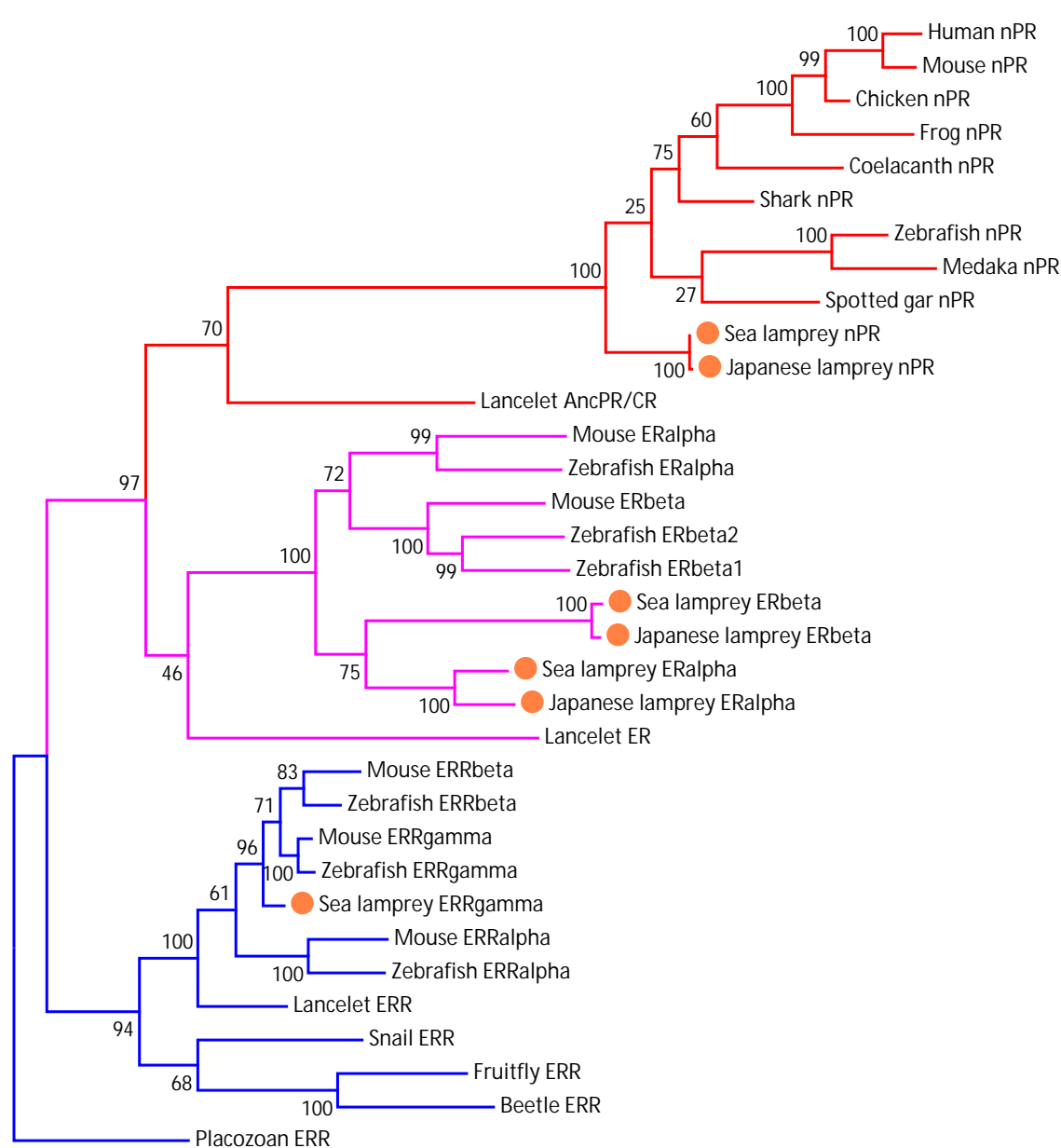

Supplement: Supplementary file 5 — A phylogenetic tree constructed by the ML method demonstrates the evolutionary relationship among nPR, ER and ERR in metazoans. (PDF 12 kb) [file 12862_2019_1463_MOESM5_ESM.pdf]

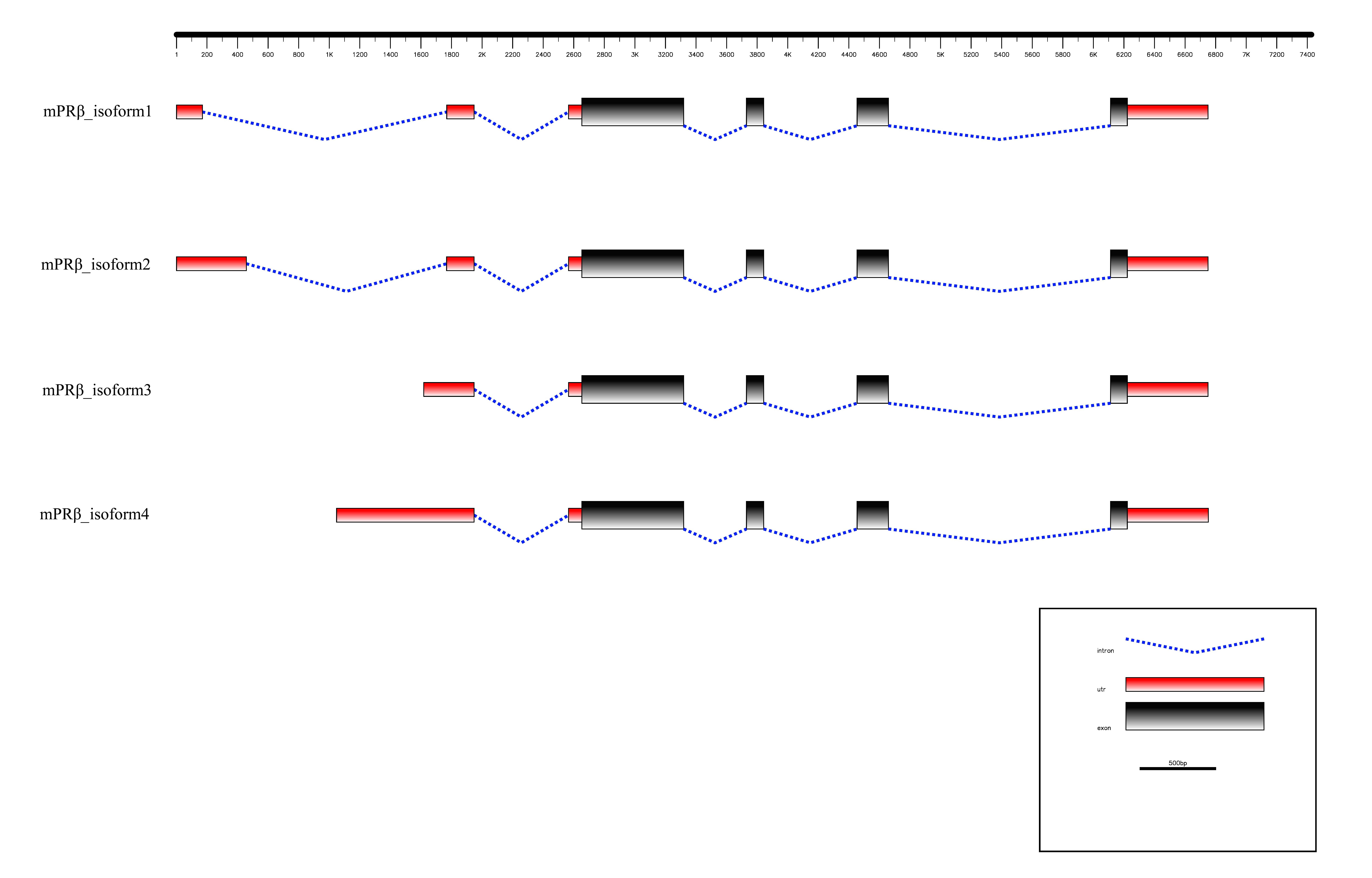

Supplement: Supplementary file 7 — Gene structure of four transcript isoforms of sea lamprey mPRβ. (JPG 1194 kb) [file 12862_2019_1463_MOESM7_ESM.jpg]

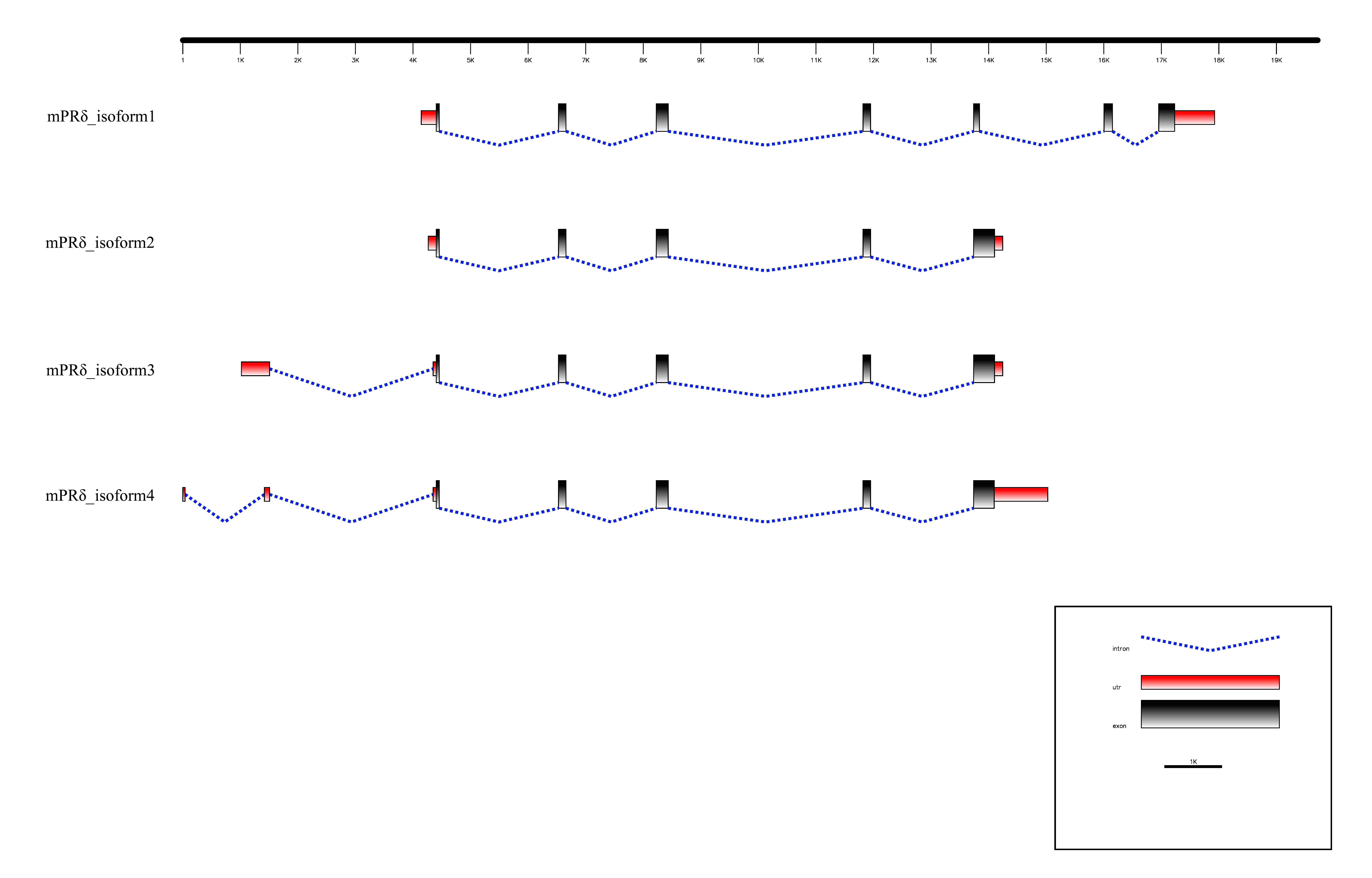

Supplement: Supplementary file 8 — Gene structure of four transcript isoforms of sea lamprey mPRδ. (JPG 1112 kb) [file 12862_2019_1463_MOESM8_ESM.jpg]

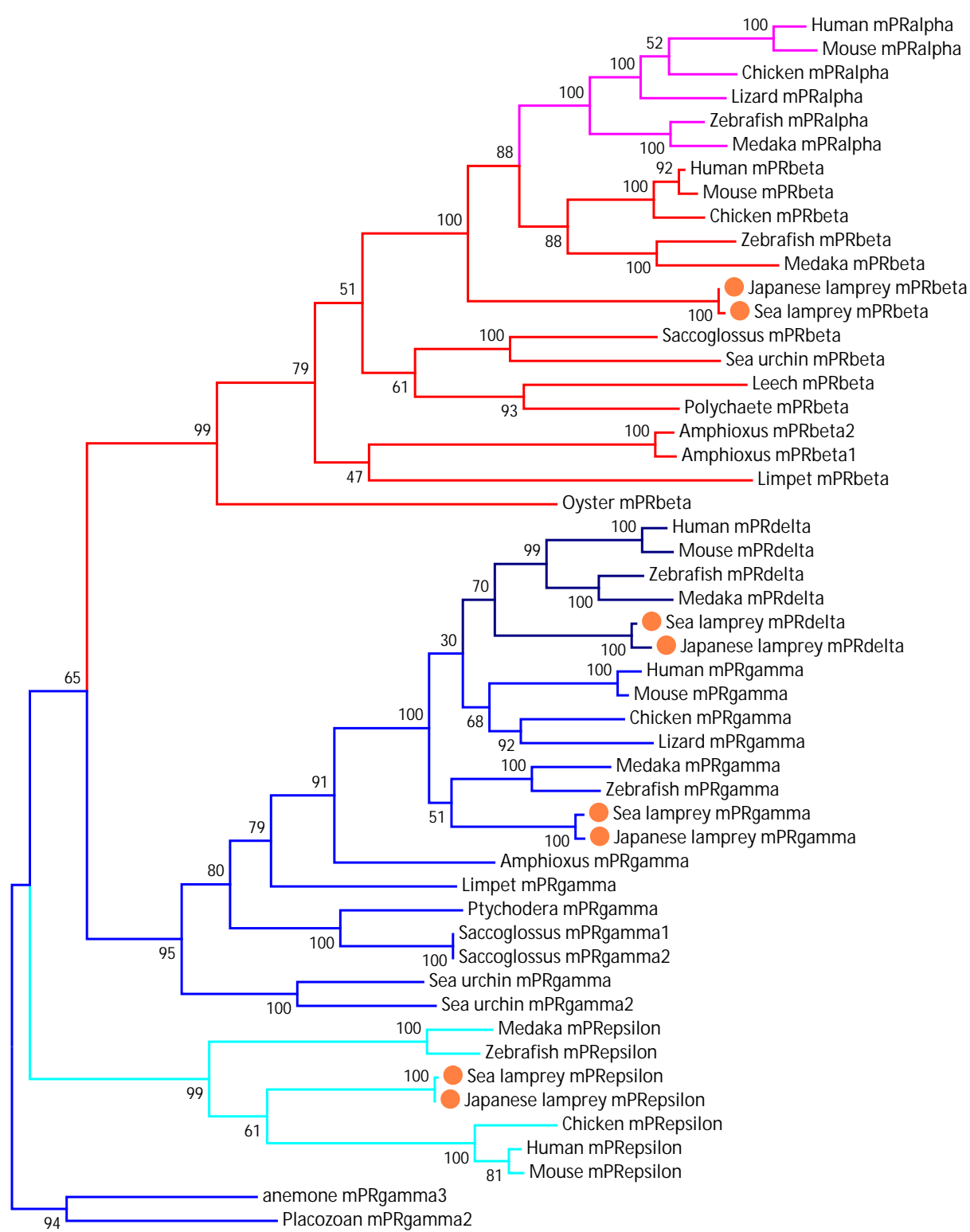

0.1

Supplement: Supplementary file 9 — A phylogenetic tree constructed by the ML method demonstrates the evolutionary relationship among five members of mPR gene family in metazoans. (PDF 16 kb) [file 12862_2019_1463_MOESM9_ESM.pdf]

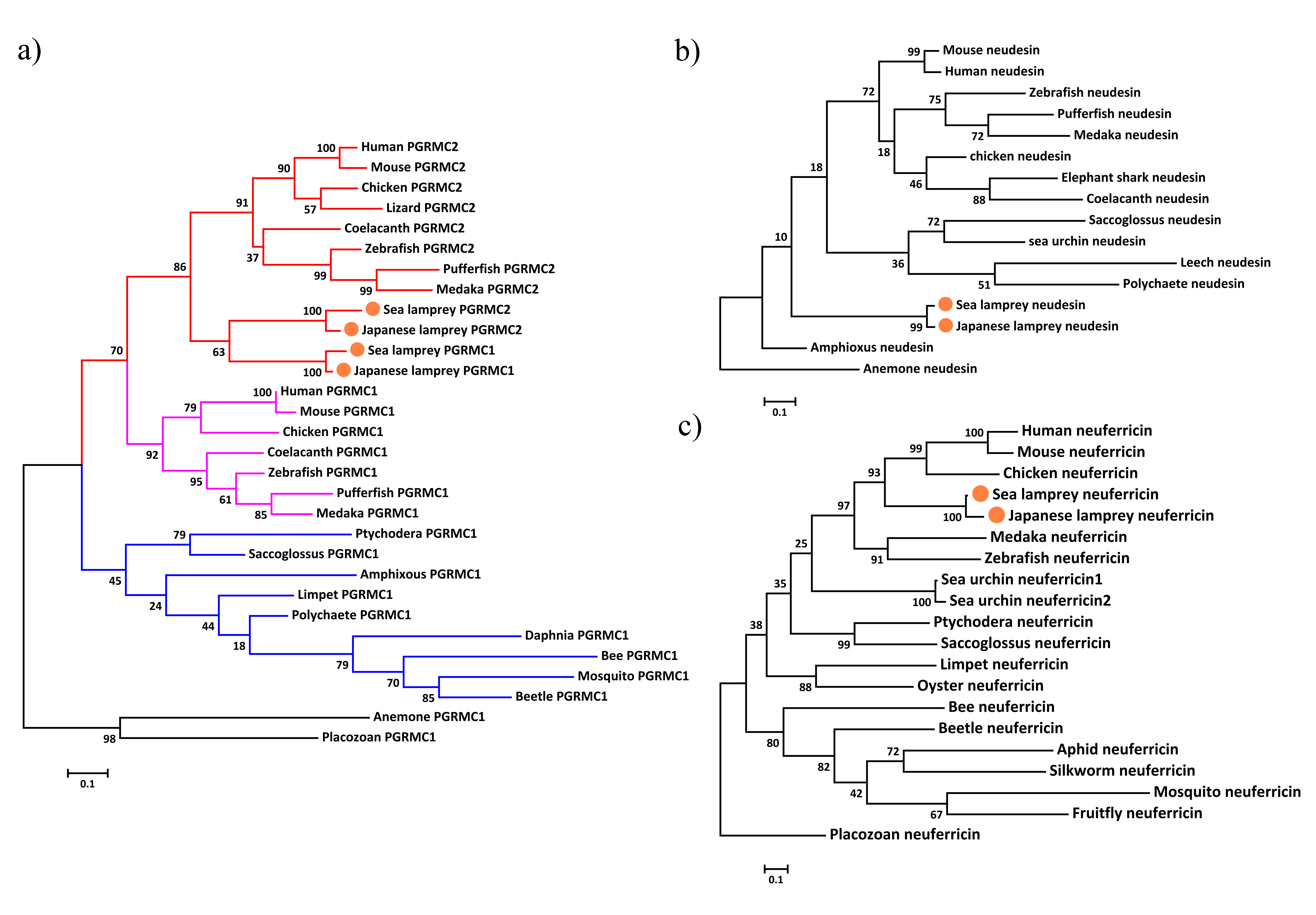

Supplement: Supplementary file 10 — Phylogenetic trees constructed by the ML method demonstrate the evolutionary relationship of PGRMC, neudesin and neuferricin in metazoans. (JPG 4300 kb) [file 12862_2019_1463_MOESM10_ESM.jpg]
