# Supplementary material for: Genomic sequence analyses of classical and non-classical lamprey progesterone receptor genes and the inference of homologous gene evolution in metazoans
Source: BMC Evol Biol. 2019 Jul 1;19:136. doi: 10.1186/s12862-019-1463-7 (PMC6604198; doi:10.1186/s12862-019-1463-7)
Supplement: Supplementary file 11 — Syntenic analysis of mPRs and MAPRs genomic sequences among human, mouse, zebrafish and lampreys. (PPTX 169 kb) [file 12862_2019_1463_MOESM11_ESM.pptx]

## Slide 1
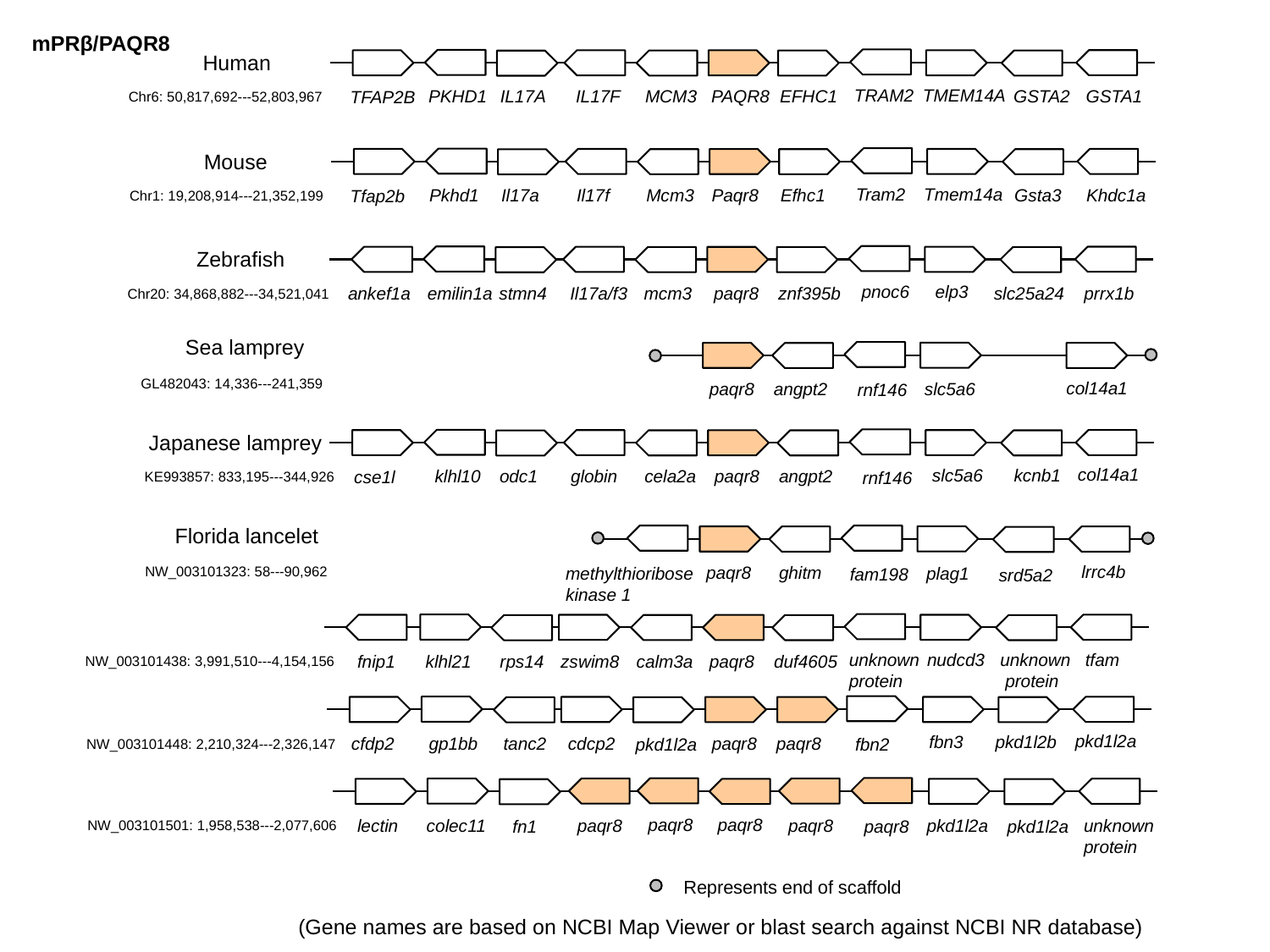

mPRβ/PAQR8
Human
TRAM2
TMEM14A
PKHD1
IL17A
MCM3
PAQR8
 EFHC1
IL17F
GSTA2
GSTA1
TFAP2B
Chr6: 50,817,692---52,803,967
Mouse
Tram2
Tmem14a
Pkhd1
Il17a
Mcm3
Paqr8
 Efhc1
Il17f
Gsta3
Khdc1a
Tfap2b
Chr1: 19,208,914---21,352,199
Zebrafish
pnoc6
elp3
emilin1a
stmn4
mcm3
paqr8
 znf395b
Il17a/f3
slc25a24
prrx1b
ankef1a
Chr20: 34,868,882---34,521,041
Sea lamprey
GL482043: 14,336---241,359
col14a1
 angpt2
slc5a6
rnf146
paqr8
Japanese lamprey
col14a1
kcnb1
slc5a6
klhl10
odc1
cela2a
paqr8
 angpt2
globin
cse1l
rnf146
KE993857: 833,195---344,926
Florida lancelet
lrrc4b
paqr8
 ghitm
NW_003101323: 58---90,962
plag1
methylthioribose
kinase 1
fam198
srd5a2
tfam
unknown
 protein
 unknown
 protein
nudcd3
klhl21
rps14
calm3a
paqr8
 duf4605
zswim8
fnip1
NW_003101438: 3,991,510---4,154,156
pkd1l2a
pkd1l2b
fbn3
gp1bb
tanc2
paqr8
 paqr8
cdcp2
cfdp2
fbn2
pkd1l2a
NW_003101448: 2,210,324---2,326,147
paqr8
paqr8
lectin
pkd1l2a
colec11
paqr8
unknown
protein
paqr8
pkd1l2a
fn1
paqr8
NW_003101501: 1,958,538---2,077,606
Represents end of scaffold
(Gene names are based on NCBI Map Viewer or blast search against NCBI NR database)

## Slide 2
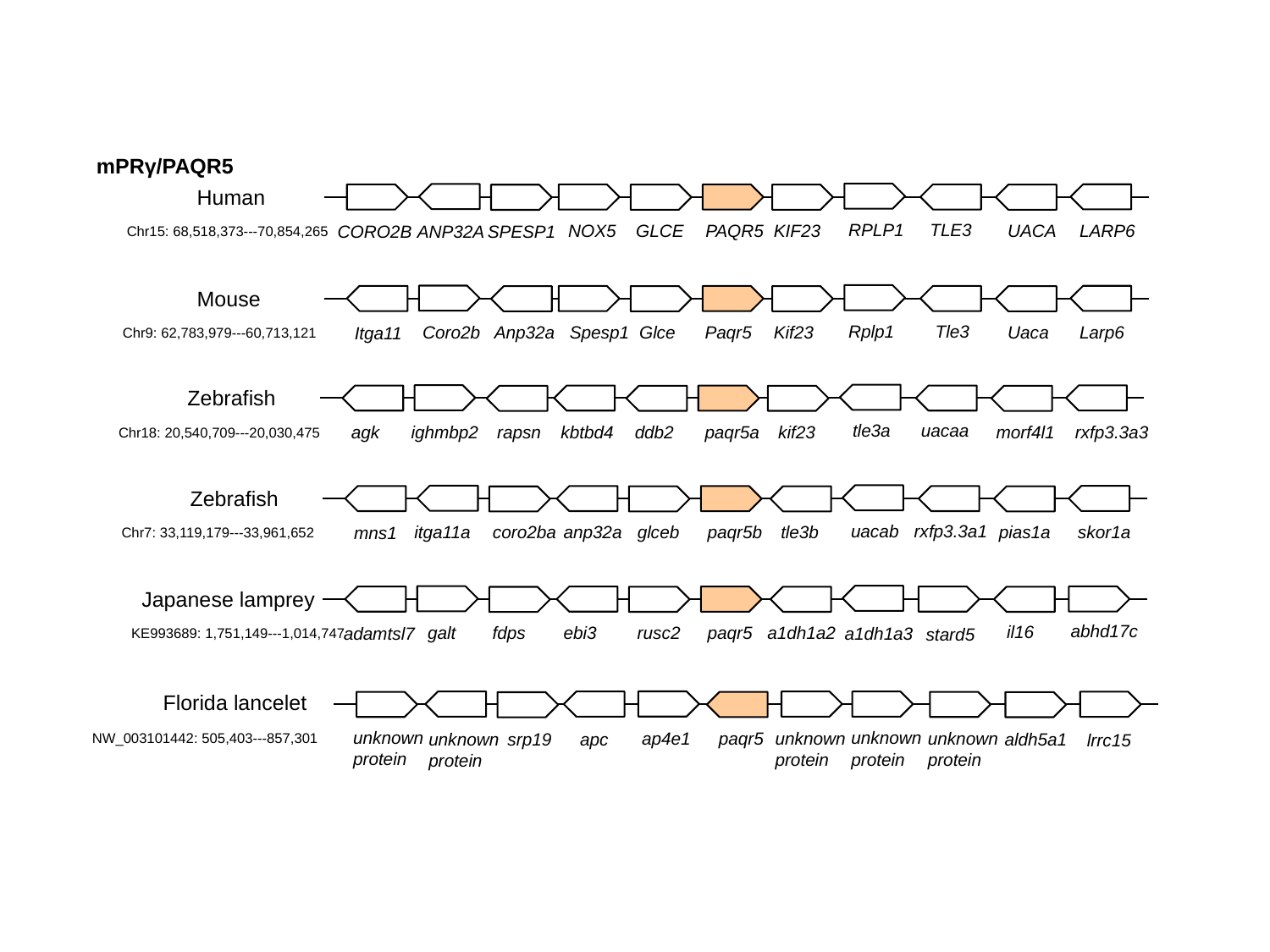

mPRγ/PAQR5
Human
RPLP1
TLE3
GLCE
PAQR5
 KIF23
NOX5
UACA
LARP6
CORO2B
ANP32A
SPESP1
Chr15: 68,518,373---70,854,265
Mouse
Rplp1
Tle3
Coro2b
Anp32a
Glce
Paqr5
 Kif23
Spesp1
Uaca
Larp6
Itga11
Chr9: 62,783,979---60,713,121
Zebrafish
tle3a
uacaa
ighmbp2
rapsn
ddb2
paqr5a
 kif23
kbtbd4
morf4l1
rxfp3.3a3
agk
Chr18: 20,540,709---20,030,475
Zebrafish
uacab
rxfp3.3a1
itga11a
coro2ba
glceb
paqr5b
 tle3b
anp32a
pias1a
skor1a
mns1
Chr7: 33,119,179---33,961,652
Japanese lamprey
abhd17c
il16
galt
fdps
rusc2
paqr5
 a1dh1a2
ebi3
adamtsl7
a1dh1a3
stard5
KE993689: 1,751,149---1,014,747
Florida lancelet
unknown
protein
unknown
protein
ap4e1
paqr5
unknown
protein
unknown
protein
unknown
protein
srp19
apc
aldh5a1
lrrc15
NW_003101442: 505,403---857,301

## Slide 3
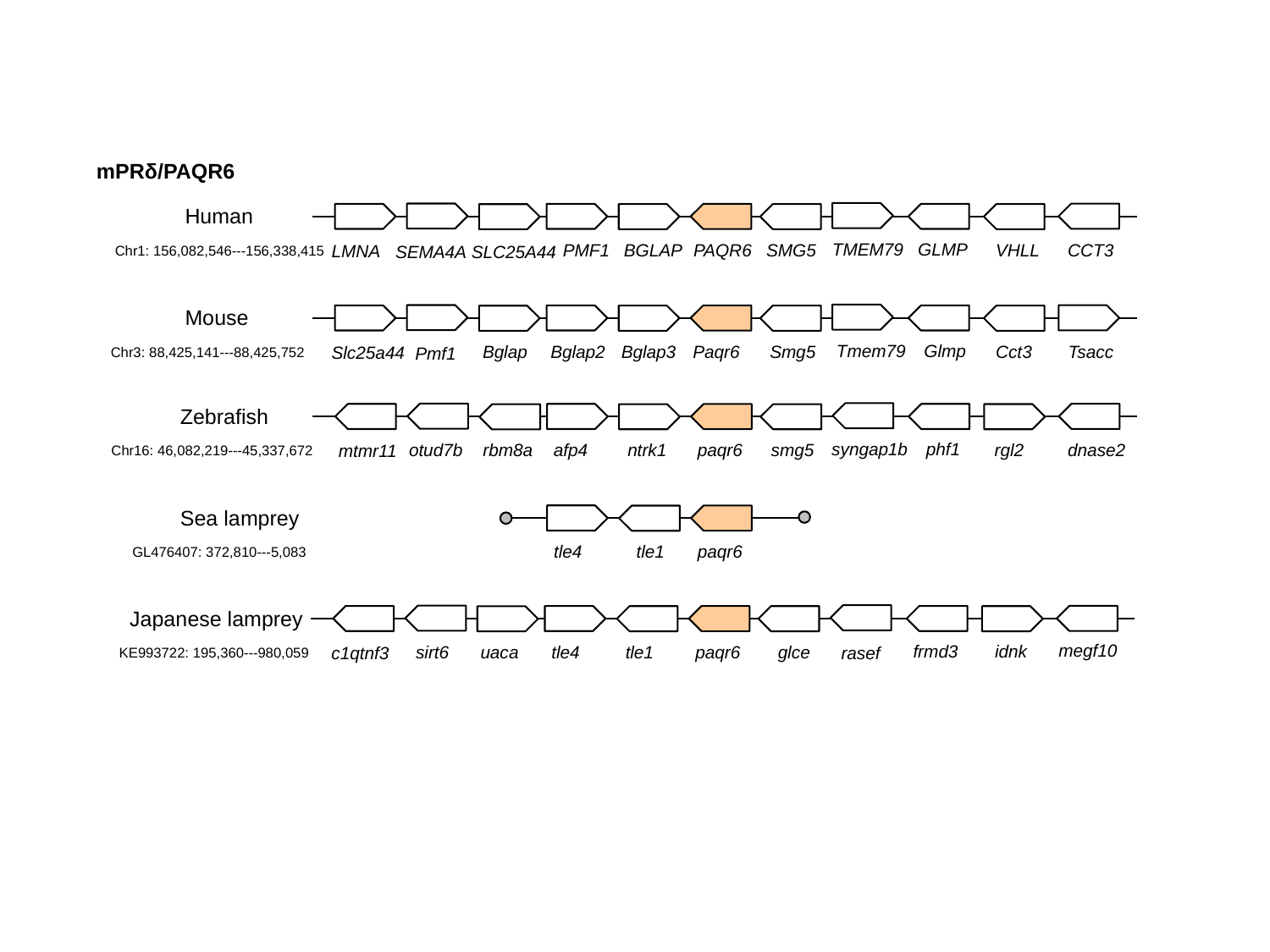

mPRδ/PAQR6
Human
TMEM79
GLMP
BGLAP
PAQR6
 SMG5
 PMF1
VHLL
CCT3
LMNA
SEMA4A
SLC25A44
Chr1: 156,082,546---156,338,415
Mouse
Tmem79
Glmp
Bglap
Bglap3
Paqr6
 Smg5
Bglap2
Cct3
Tsacc
Slc25a44
Pmf1
Chr3: 88,425,141---88,425,752
Zebrafish
syngap1b
phf1
otud7b
rbm8a
ntrk1
paqr6
 smg5
afp4
rgl2
dnase2
mtmr11
Chr16: 46,082,219---45,337,672
Sea lamprey
tle1
paqr6
tle4
GL476407: 372,810---5,083
Japanese lamprey
megf10
idnk
frmd3
sirt6
uaca
tle1
paqr6
 glce
tle4
c1qtnf3
rasef
KE993722: 195,360---980,059

## Slide 4
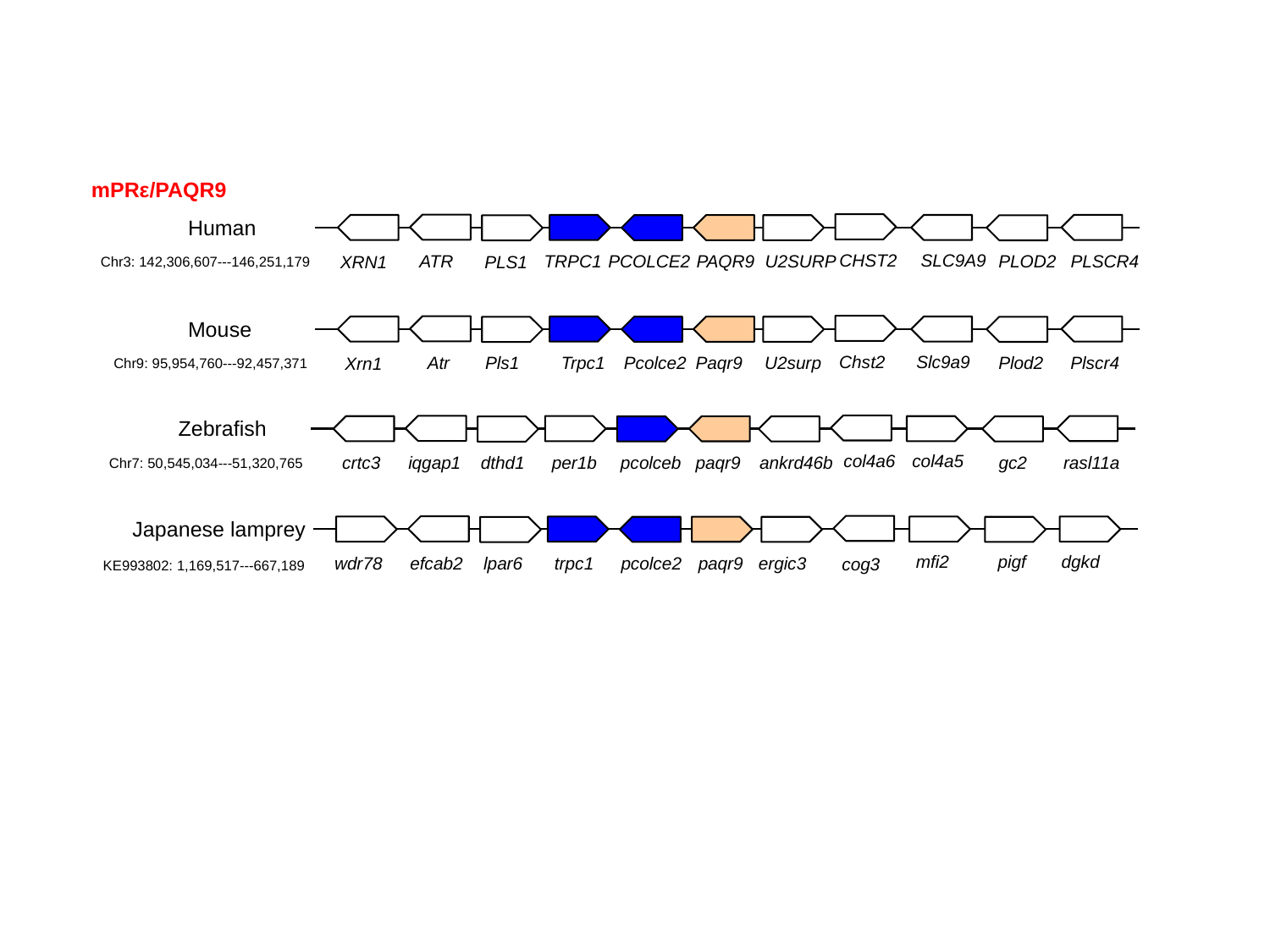

mPRε/PAQR9
Human
CHST2
SLC9A9
ATR
PCOLCE2
PAQR9
 U2SURP
TRPC1
PLOD2
PLSCR4
XRN1
PLS1
Chr3: 142,306,607---146,251,179
Mouse
Chst2
Slc9a9
Atr
Pls1
Pcolce2
Paqr9
 U2surp
Trpc1
Plod2
Plscr4
Xrn1
Chr9: 95,954,760---92,457,371
Zebrafish
col4a6
col4a5
iqgap1
dthd1
pcolceb
paqr9
 ankrd46b
per1b
gc2
rasl11a
crtc3
Chr7: 50,545,034---51,320,765
Japanese lamprey
dgkd
pigf
mfi2
efcab2
lpar6
pcolce2
paqr9
 ergic3
trpc1
wdr78
cog3
KE993802: 1,169,517---667,189

## Slide 5
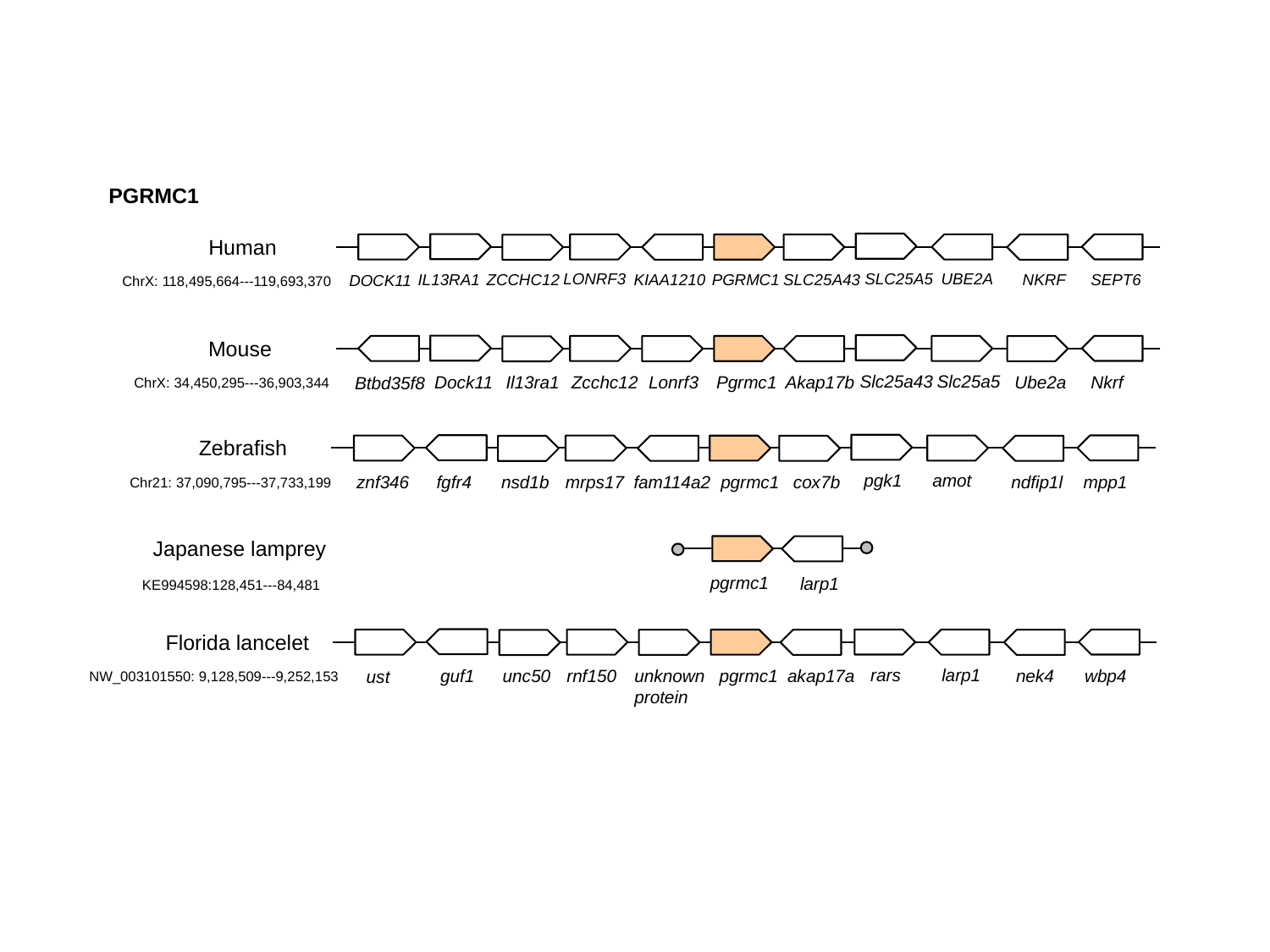

PGRMC1
Human
 SLC25A43
SLC25A5
UBE2A
LONRF3
IL13RA1
ZCCHC12
KIAA1210
PGRMC1
NKRF
SEPT6
DOCK11
ChrX: 118,495,664---119,693,370
Mouse
Slc25a43
Slc25a5
Dock11
Il13ra1
Lonrf3
Pgrmc1
 Akap17b
Zcchc12
Ube2a
Nkrf
Btbd35f8
ChrX: 34,450,295---36,903,344
Zebrafish
pgk1
amot
fgfr4
nsd1b
fam114a2
pgrmc1
 cox7b
mrps17
ndfip1l
mpp1
znf346
Chr21: 37,090,795---37,733,199
Japanese lamprey
pgrmc1
 larp1
KE994598:128,451---84,481
Florida lancelet
rars
larp1
guf1
unc50
unknown
protein
pgrmc1
 akap17a
rnf150
nek4
wbp4
ust
NW_003101550: 9,128,509---9,252,153

## Slide 6
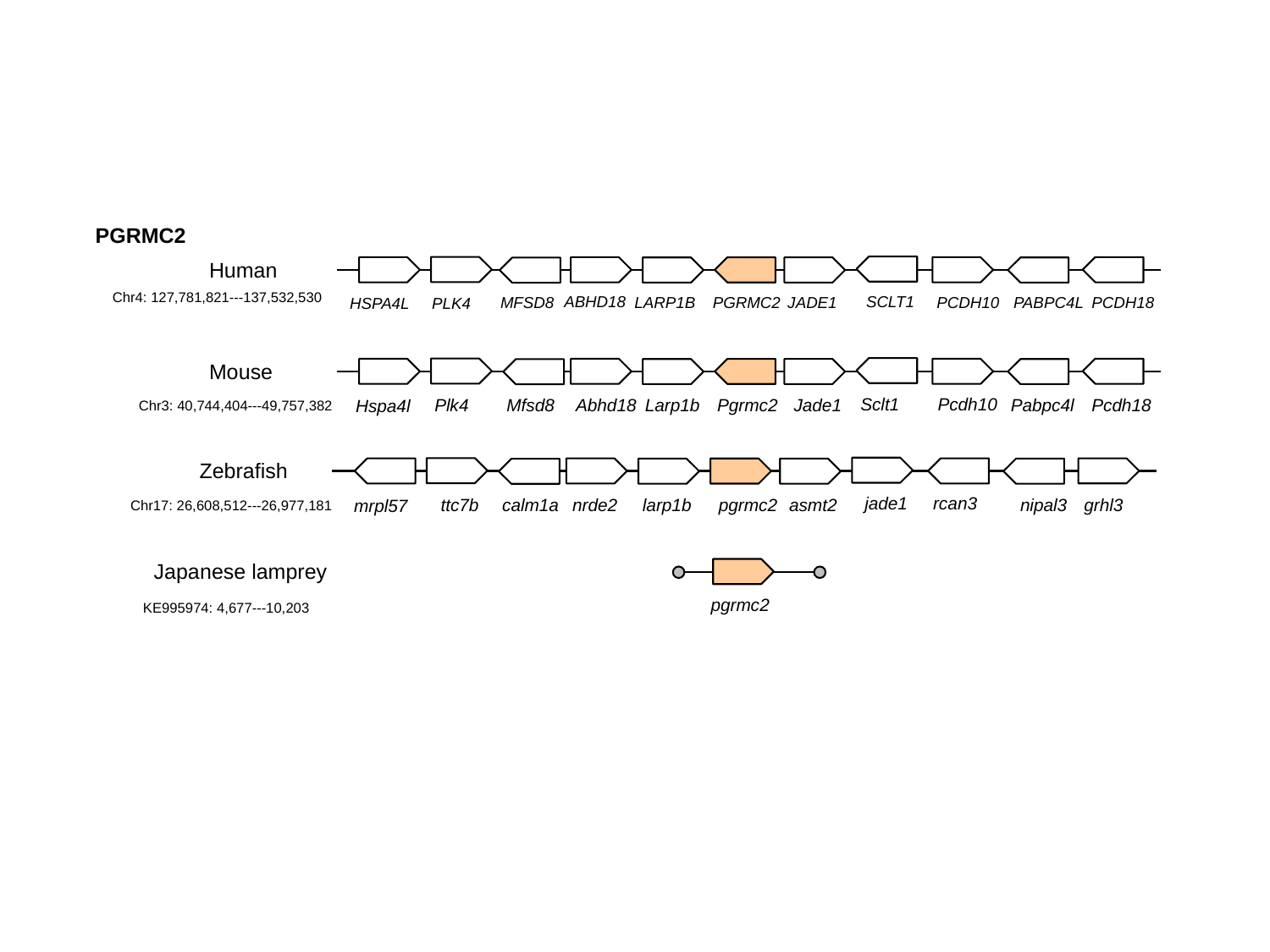

PGRMC2
Human
Chr4: 127,781,821---137,532,530
 JADE1
SCLT1
ABHD18
PCDH10
MFSD8
LARP1B
PGRMC2
PABPC4L
PCDH18
HSPA4L
PLK4
Mouse
Sclt1
Pcdh10
Plk4
Mfsd8
Larp1b
Pgrmc2
 Jade1
Abhd18
Pabpc4l
Pcdh18
Hspa4l
Chr3: 40,744,404---49,757,382
Zebrafish
jade1
rcan3
ttc7b
calm1a
larp1b
pgrmc2
asmt2
nrde2
nipal3
grhl3
mrpl57
Chr17: 26,608,512---26,977,181
Japanese lamprey
pgrmc2
KE995974: 4,677---10,203

## Slide 7
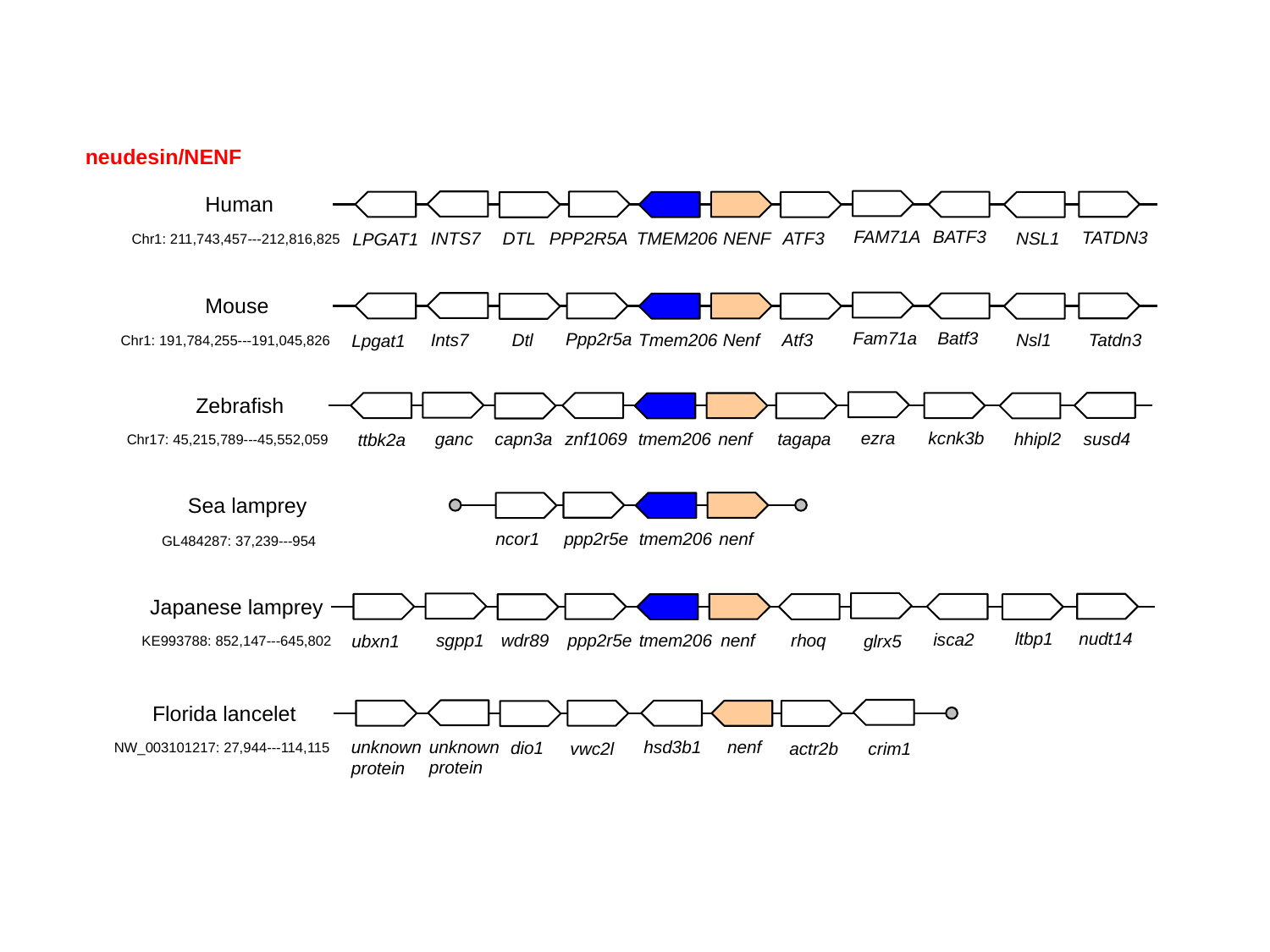

neudesin/NENF
Human
FAM71A
BATF3
TATDN3
INTS7
DTL
TMEM206
NENF
 ATF3
PPP2R5A
NSL1
LPGAT1
Chr1: 211,743,457---212,816,825
Mouse
Fam71a
Batf3
Ppp2r5a
Ints7
Dtl
Tmem206
 Nenf
 Atf3
Nsl1
Tatdn3
Lpgat1
Chr1: 191,784,255---191,045,826
Zebrafish
ezra
kcnk3b
ganc
capn3a
tmem206
nenf
 tagapa
znf1069
hhipl2
susd4
ttbk2a
Chr17: 45,215,789---45,552,059
Sea lamprey
ncor1
tmem206
nenf
ppp2r5e
GL484287: 37,239---954
Japanese lamprey
nudt14
ltbp1
isca2
sgpp1
wdr89
tmem206
nenf
 rhoq
ppp2r5e
ubxn1
glrx5
KE993788: 852,147---645,802
Florida lancelet
unknown
protein
hsd3b1
nenf
dio1
 actr2b
vwc2l
crim1
NW_003101217: 27,944---114,115
unknown
protein

## Slide 8
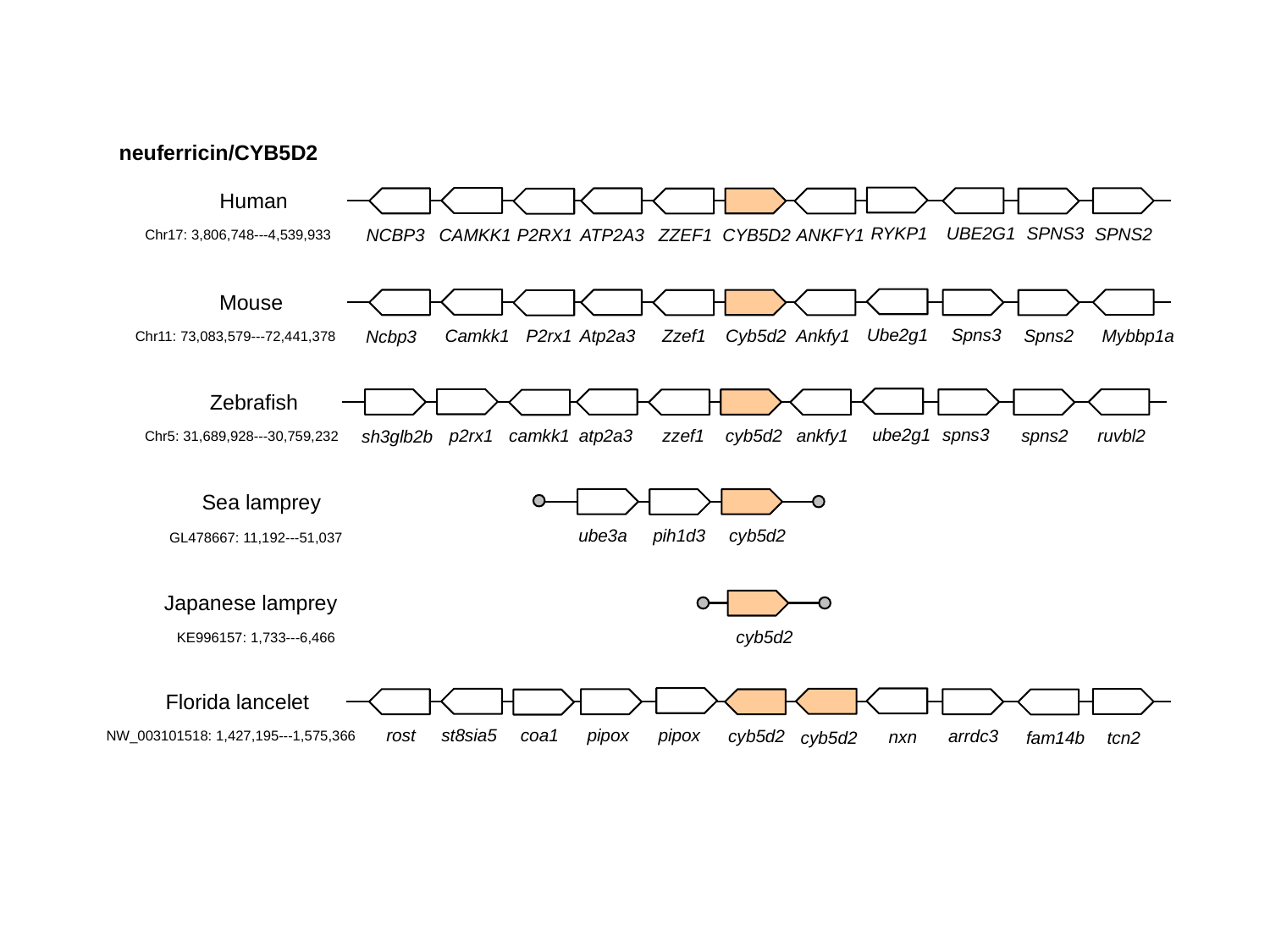

neuferricin/CYB5D2
Human
RYKP1
UBE2G1
SPNS3
SPNS2
CAMKK1
ATP2A3
ZZEF1
CYB5D2
 ANKFY1
NCBP3
P2RX1
Chr17: 3,806,748---4,539,933
Mouse
Ube2g1
Spns3
Atp2a3
Camkk1
P2rx1
Zzef1
 Cyb5d2
 Ankfy1
Spns2
Mybbp1a
Ncbp3
Chr11: 73,083,579---72,441,378
Zebrafish
ube2g1
spns3
p2rx1
camkk1
zzef1
cyb5d2
 ankfy1
atp2a3
spns2
ruvbl2
sh3glb2b
Chr5: 31,689,928---30,759,232
Sea lamprey
ube3a
pih1d3
cyb5d2
GL478667: 11,192---51,037
Japanese lamprey
cyb5d2
KE996157: 1,733---6,466
Florida lancelet
 rost
st8sia5
coa1
pipox
pipox
cyb5d2
arrdc3
nxn
 cyb5d2
fam14b
tcn2
NW_003101518: 1,427,195---1,575,366
